# Identification of tumor-associated cassette exons in human cancer through EST-based computational prediction and experimental validation

**DOI:** 10.1186/1476-4598-9-230

**Published:** 2010-09-02

**Authors:** Alessio Valletti, Anna Anselmo, Marina Mangiulli, Ilenia Boria, Flavio Mignone, Giuseppe Merla, Vincenzo D'Angelo, Apollonia Tullo, Elisabetta Sbisà, Anna Maria D'Erchia, Graziano Pesole

**Affiliations:** 1 University of Bari, Dipartimento di Biochimica e Biologia Molecolare "E. Quagliariello", via Orabona, 4, Bari 70126, Italy; 2 University of Milan, Dipartimento di Scienze Biomolecolari e Biotecnologie, via Celoria 26, Milan 20133, Italy; 3 University of Milan, Dipartimento di Chimica strutturale e Stereochimica Inorganica, via Venezian, 12, Milan 20133, Italy; 4 Laboratorio di Genetica Medica, IRCCS Casa Sollievo della Sofferenza, 71013 San Giovanni Rotondo, Italy; 5 Dipartimento di Neuroscienze, Divisione di Neurochirurgia, IRCCS Casa Sollievo della Sofferenza, 71013 San Giovanni Rotondo, Italy; 6 Istituto Tecnologie Biomediche del Consiglio Nazionale delle Ricerche, sede di Bari, via Amendola 122/D, Bari 70126, Italy; 7 Istituto Biomembrane e Bioenergetica del Consiglio Nazionale delle Ricerche, via Amendola 165/A, 70126 Bari, Italy

## Abstract

**Background:**

Many evidences report that alternative splicing, the mechanism which produces mRNAs and proteins with different structures and functions from the same gene, is altered in cancer cells. Thus, the identification and characterization of cancer-specific splice variants may give large impulse to the discovery of novel diagnostic and prognostic tumour biomarkers, as well as of new targets for more selective and effective therapies.

**Results:**

We present here a genome-wide analysis of the alternative splicing pattern of human genes through a computational analysis of normal and cancer-specific ESTs from seventeen anatomical groups, using data available in AspicDB, a database resource for the analysis of alternative splicing in human. By using a statistical methodology, normal and cancer-specific genes, splice sites and cassette exons were predicted *in silico*. The condition association of some of the novel normal/tumoral cassette exons was experimentally verified by RT-qPCR assays in the same anatomical system where they were predicted. Remarkably, the presence *in vivo *of the predicted alternative transcripts, specific for the nervous system, was confirmed in patients affected by glioblastoma.

**Conclusion:**

This study presents a novel computational methodology for the identification of tumor-associated transcript variants to be used as cancer molecular biomarkers, provides its experimental validation, and reports specific biomarkers for glioblastoma.

## Background

Alternative splicing (AS) is a pivotal regulation mechanism allowing the expansion of the genome expression potential through the generation of multiple transcripts from a single gene. Indeed, over 90% of multi-exon genes undergo AS [1] generating on average a tenfold expansion of the transcriptome complexity [2]. Alternatively spliced exons have splice sites that can be specifically recognized depending on tissue specificity, developmental stage, external stimuli, cellular stress, or even pathological conditions [3]. So the presence or the expression level of specific splice variants can be indicative of a pathological or physiological condition or even be the cause of a disease. Recent studies demonstrated that alternative isoforms can be linked to many pathologies among which cancer, and play important roles in their etiopathogenesis [4]. Indeed, changes in the splicing pattern of a gene may affect different steps in the life of a cell (e.g. cell growth, adhesion, migration, invasion and cell death) eventually leading to subsequent tumor formation [5]. Several evidences of the association between alternative splicing and cancer have been reported. For example, it has been shown that about half of all active alternative splicing events in ovarian and breast tissues are altered in tumors, resulting in a massive tissue type-specific reprogramming of alternative splicing [6]. These alterations seems to be linked to the RNA binding protein FOX2 whose expression is downregulated in ovarian cancer and whose splicing results extensively changed in breast cancer samples. More recently, it has been reported that the presence of an alternatively spliced isoform of the receptor for factor VII/VIIa, named Tissue Factor (TF), is a prognostic marker in patients with non-small cell lung cancer [7]. Indeed, this soluble isoform of TF lacking exon 5 that encodes the transmembrane domain has been detected in various cancerous tissues and several studies suggested that its expression promotes tumor growth and is associated with increased tumor cell proliferation and angiogenesis in vivo [8]. Association of splicing with diseases does not necessarily imply that alternative splicing is a direct cause of cancer: aberrant splicing may be consequence of indirect effects produced by the disease-induced stress or may be caused by the general misregulation of cellular functions [9]. Nonetheless, the identification of a robust association between diseases and specific splicing patterns could be very useful to define signatures that can be used as diagnostic or prognostic indicators, allowing to obtain medically relevant information such as tumor stage and profiles [10,11].

The availability of an increasing number of genome and transcript sequences, including Expressed Sequence Tag sequences (ESTs) and more recently RNA-Seq data, provides an essential information source for the computational detection of the alternative splicing pattern of genes [1,2,12-15].

Currently, dbEST comprises more than 62 million ESTs from several hundred organisms, the highest numbers for individual organisms being over eight million for human [16]. Several bioinformatic tools have been developed for genome-wide detection of alternative splicing based on pairwise alignments of cDNA and genomic sequences [17,18]. To improve the prediction accuracy, we developed a new tool, we named ASPic. ASPic implements an optimization strategy that performing a multiple alignment of all available transcript data (including full-length cDNA and EST sequences) to the genomic sequence detects the intron set that minimizes the number of splicing sites. It also generates, through a DAG-based combinatorial procedure, the minimal set of non-mergeable transcript isoforms compatible with the detected splicing events [19]. The reliability of splicing isoforms detected by ASPic has been recently established through a comparative assessment [20].

EST sequences have linked information about their source sample (commonly including tissue, cancer, gender, and developmental stage as well as organism information) [21]. In particular, 45% of EST data are collected from cancer tissues thus providing an invaluable source of information to study association of alternative splicing with cancer. Furthermore, the longer read length of EST data with respect to those obtainable from current next generation sequencing platforms may provide relevant information on exon connectivity, thus allowing a more accurate reconstruction of full length alternative transcripts. Assuming that the expression level of a splice variant in a certain source sample is roughly proportional to the number of related ESTs in the cDNA library [16], we may provide a qualitative and quantitative association of the detected splice sites according to tissue-specificity and tumor status.

Recently, several studies reported the identification of cancer specific variants by combining the computational analysis of the EST data with experimental validations [22-24].

The availability of the unprecedented repertoire of 319,092 alternative splicing isoforms from 18,174 genes collected in ASPicDB [2] predicted by a comprehensive analysis of EST data included in the Unigene database [25] prompted us to develop a reliable strategy for the identification of cancer-associated isoforms. In particular, we focused our attention on the identification of alternative normal- and tumor-specific cassette exons which are particularly suitable as diagnostic or prognostic cancer biomarkers or as therapeutic targets. Remarkably, we newly discovered several cassette exons with a significant differential expression in normal and cancer conditions. For some of them, we carried out an experimental validation by RT-qPCR assays on commercial RNA from the same anatomical system where they were predicted. Considerably, we assessed the biomarker suitability of some cassette exons, found differentially expressed in the nervous system, in glioblastoma patients. Our results provide support to our computational analysis and suggest that the relative ratio between normal and tumor alternative transcripts may represent a promising tumor biomarker.

## Methods

### Data source and computational analysis

**Figure 1:**
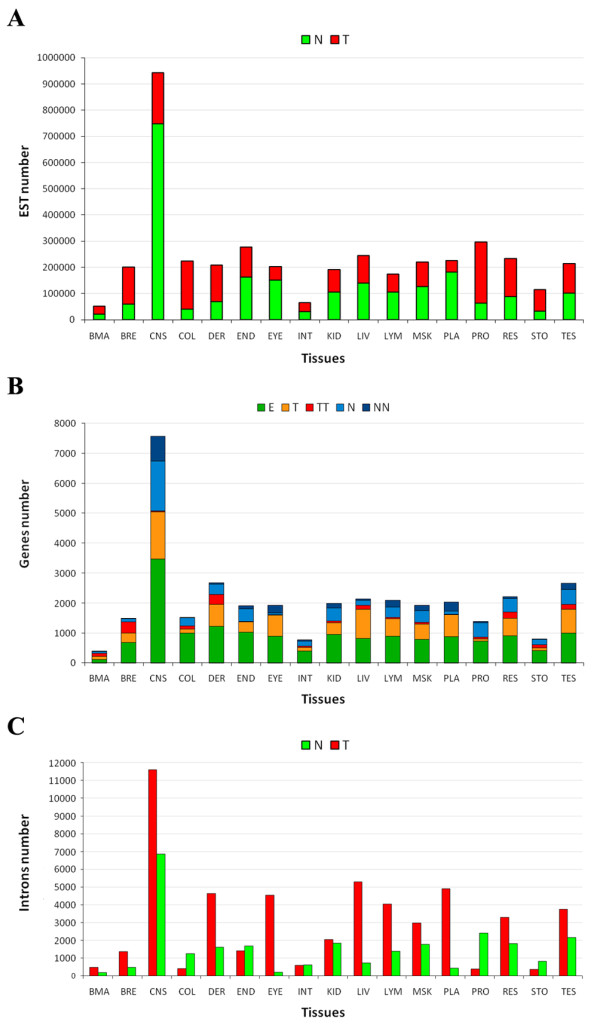
**Genes and introns classification based on ESTs data analyses**. **(A) **Number of available ESTs for each tissue, classified as normal (N) or tumoral (T), according to the CGAP classification. **(B) **Number of genes analyzed for each tissue, classified on the base of their expression in the normal (N and NN) and tumoral (T and TT) status and of comparable expression in both conditions (E). **(C) **Number of tumoral (T) and normal (N) specific introns for each tissue. BMA: Bone marrow, BRE: Breast, CNS: Central nervous system (e.g. brain); COL: Colon; DER: Skin; END: Endocrine system; EYE: Eye; INT: Gastrointestinal tract; KID: Kidney; LIV: Liver; LYM: Lymphoreticular system; MSK: Musculoskeletal system; PLA: Placenta; PRO: Prostate; RES: Respiratory System; STO: Stomach; TES: Testis

We used as data source for the detection of cancer specific isoforms the ASPicDB database [2]. For all human genes, ASPicDB provides information on all putative splicing variants, as well as on the supporting ESTs of each specific splice site. ESTs have been classified in 17 anatomical systems (see Figure 1) matching their library information and following the nomenclature of the "Cancer Genome Anatomy Project" http://www.ncbi.nlm.nih.gov/ncicgap/[26]. We considered only those anatomical systems for which at least 30,000 ESTs were available in both the normal and tumor conditions.

To evaluate if the ESTs count between tumoral and normal tissues was significantly different, we computed upper-tail p-values (< 0.05) based upon the hypergeometric distribution [16,27], also adopting the Bonferroni correction to address the problem of multiple comparisons. Given the large size of libraries and the small size of the sample (count of ESTs) we did not assume a normal distribution (like in the Z-test used by Wang et al. [24]) but we chose a nonparametric method. We classified in the same way splicing sites.

The analysis of Gene Ontology (GO) annotation was performed using terms related to biological processes [28]. Beside the Gene Ontology annotation available for each human gene, we also assigned every parent term up to the root discarding GO terms represented on genome scale by less than 50 genes.

Ontology terms assigned to genes classified as over-represented in tumor condition (T/TT classes) or in physiological condition (N/NN or E classes) were then identified and compared to their occurrence in the whole genome set. Enriched GO classes were identified by using Fisher's exact test with a P≤0.01 cut off. The Ingenuity Pathway analysis tool [29] has been used to detect specific functional feature enrichments in selected gene sets.

### RT-qPCR experiments

Total RNA from human normal (skeletal muscle and brain) and tumor tissues (rhabdomyosarcoma and neuroblastoma) was purchased from commercial sources (Ambion and Asterand, respectively). Glioma samples were obtained from the Neurosurgery Department, IRCCS Casa Sollievo della Sofferenza (San Giovanni Rotondo, Italy). Informed consent was obtained for all patients before the surgery as approved by the ethics board. All tumors were histologically classified as glioblastoma multiforme (GBM, WHO grade IV). Samples were collected immediately after surgical resection and total RNA was extracted after having established primary glioma cell lines, with RNeasy Mini Kit (Qiagen) accordingly to the manufacturer's instructions.

cDNA synthesis was performed from 1 μg of total RNA using QuantiTect^® ^Reverse Transcription kit (Qiagen).

Primers were designed with Primer3 software to detect all splicing events determining the inclusion/exclusion of the predicted tumoral or normal cassette exons. For STRADA, PCNP and NAP1L1 genes, for which computational analysis identified two associated cassette exons, primers were designed to amplify both exons or to detect their simultaneous absence. For SLC25A3 gene, where mutually exclusive exons were predicted, primers were designed on specific exon junctions resulting from alternative splicing events occurred. The sequences of all primers are listed in Additional File 1.

1 μl of each cDNA (dilution 1:10) was used as template in qPCR assays, performed in triplicate on ABI PRISM 7900HT (Applied Biosystems) using the QuantiTect^® ^SYBR Green PCR Master Mix (Qiagen). Amplification parameters were as follows: hot start at 95°C for 15 min; 50 cycles of amplification (94°C for 15 sec, 62°C for 30 sec, 72°C for 30 sec); dissociation curve step (95°C for 15 sec, 60°C for 15 sec, 95°C for 15 sec). Fluorescence raw data were exported from the SDS 2.2.1 software (Applied Biosystems) and analyzed with the DART-PCR Excel workbook [30]. For each tissue, the relative expression ratio (rER) of transcripts with the assayed cassette exons respect to those lacking them was calculated applying the following formula: (1 + E_(w/o exon)_)^Ct(w/o exon)^/(1 + E_(with exon)_)^Ct(with exon)^, where E is the average amplification efficiency calculated by DART-PCR for each primers pair and Ct is the average Ct obtained for the presence of the cassette exon or for its absence. No normalization was needed because comparisons were carried out between targets in the same sample. The data shown are the average from at least three independent experiments.

## Results

Genes and introns classification and prediction of alternative splicing transcript variants

To carry out our genome-wide analysis of cancer related splicing isoforms, we considered all data collected in our ASPicDB database [2] including over 18,000 genes and 300,000 alternative transcripts.

As depicted in Figure 1A, the number of available ESTs was quite heterogeneous for the different tissues. More than 900,000 ESTs derive from central nervous system (CNS) and - on average - about 210,000 ESTs are available for all the other considered tissues. The ratio between tumoral and normal ESTs is close to one in tissues like gastrointestinal tract (INT), kidney (KID) and testis (TES), as the number of available ESTs in the two conditions is almost the same. Tissues like colon (COL) and prostate (PRO) are enriched by sequences obtained from tumoral libraries, conversely placenta (PLA) and central nervous system (CNS) contain more ESTs obtained from normal tissues.

The overall expression of each gene (represented by at least 10 ESTs) has been evaluated in all the considered anatomical groups and in both the normal and neoplastic status. For each gene - and for each tissue - we compared the number of ESTs from normal and neoplastic samples. Genes were grouped into five categories: genes exclusively expressed in normal condition (NN); genes exclusively expressed in neoplastic condition (TT); genes over-expressed in normal condition (N); genes over-expressed in neoplastic condition (T); genes with comparable expression in both normal and neoplastic conditions (E). Figure 1B summarizes, for each tissue, the distribution of genes in the five different classes. As expected, the number of classifiable genes in each tissue - although relatively small compared to the total number of analyzed genes - reflects the abundance of available ESTs. As a matter of fact, the highest number of classified genes (> 7000) belongs to CNS. On average, 40% of genes do not show a statistically significant differential expression in the two conditions (class E). The remaining 60% of genes are distributed among the 4 different classes (TT, T, NN, N). Bone marrow (BMA), breast (BRE), skin (DER), liver (LIV) and placenta (PLA) show a significant bias toward genes over-expressed in tumors (T and TT). Conversely, central nervous system (CNS) and prostate (PRO) are enriched in genes mainly expressed in normal tissues (N and NN). The genes exclusively expressed in the neoplastic condition in the different tissues, which could be considered tumoral markers in a specific tissue, are listed in Additional File 2.

In order to assess if genes mainly expressed in tumor cells with respect to genes resulting mainly or equally expressed in normal tissues were enriched in specific functional categories, we performed a statistic analysis using the Gene Ontology annotation. The occurrence of each GO term in cancer associated genes (T and TT classes) and in genes classified as N, NN or E was compared to its occurrence in the whole genes set thus identifying significantly over-represented ontologies by applying Fisher's exact test. Additional File 3 lists the top 10 most frequent terms in the two genes sets. Our results, not surprisingly, show that genes mainly expressed in normal tissues are related to general metabolic processes, while genes associated to cancer show - as expected - a sizable enrichment of processes related to cell proliferation, apoptosis and cell cycle.

In order to identify cancer or normal specific introns, we considered in our analysis all transcript-supported splices of genes belonging to the T, N and E classes, excluding genes belonging to the TT and NN classes. As shown in Figure 1C and detailed in Additional File 4, the majority of classified splicing events belongs to CNS with 18,436 classified introns, due to the higher number of ESTs available for CNS (Figure 1A). With few exceptions (e.g. colon, prostate and stomach), a general prevalence of tumor associated introns can be observed, with eye and placenta showing a noticeably 96% and 92% of tumor associated introns. This pattern is not related to the differential abundance of normal and tumor specific ESTs, as shown in Figure 1A. Indeed, for colon, prostate, and stomach, tumor associated-ESTs largely outnumber normal ones (Figure 1A).

By using the methodology described in Methods, we classified 26,974 splice sites as tumor- (T or TT) and 21,120 as normal- (N or NN) specific from 9513 genes. Given the variable number of aligned ESTs for each gene and their uneven coverage across its length, each splice site was classified in one or more tissues. For example, only three splices were classifiable in all 17 tissues considered and 991 in at least 10 tissues. We then investigated the classification concordance for introns labeled in five or more tissues. In most of cases (2993 introns, 62.2%) we detected an heterogeneous classification where the same intron was classified normal in some tissues and tumoral in others. For example, the first intron of PPP2R1A gene was exclusively expressed in the normal END tissue and in the tumoral BMA tissue (see Additional File 5). The remaining cases were homogeneously tumoral (1610, 33.5%) or normal (205, 4.3%).

We finally evaluated the classified introns on the basis of the spliceosomal machinery by which they are recognized (U2 or U12 spliceosome) without detecting any significant bias (data not shown).

Identification of alternative Tumoral/Normal introns and cassette-exons and mutually exclusive exons

The further step of our analysis was aimed to the identification of alternative N- and T-specific introns, based on their overlapping coordinates. Table 1 summarizes - for each considered tissue - the number of genes for which we were able to identify at least one tumor-associated splicing event not compatible (i.e. alternative) with a normal-associated one. The number of alternative tumoral and normal introns is also reported. We identified the highest number of genes [22] in the CNS, fitting the above mentioned criterion, related to the high number of available ESTs for this tissue.

**Table 1:** Number of genes with normal and tumoral introns and exons for each tissue

					Cassette Exons	
					
Tissue	Number of Genes	Number of Introns	Normal introns	Tumoral introns	T	N
CNS	22	92	59	33	9	34
DER	4	16	5	11	8	1
EYE	2	7	3	4	2	1
KID	1	3	1	2	1	0
LIV	1	3	2	1	0	1
LYM	4	17	9	8	4	4
MSK	7	24	13	11	3	6
PLA	2	6	3	3	1	1
RES	2	12	5	7	4	2
TES	5	17	10	8	2	4
*Total*	***50***	***197***	***109***	***88***	***34***	***54***

**Figure 2:**
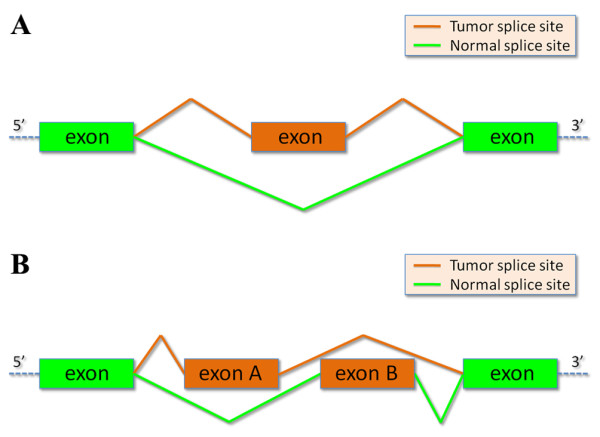
**Cassette and mutually exclusive exons**. Schematic representation of a tumoral cassette exon **(A) **and mutually exclusive exons **(B)**. Tumoral cassette exon or mutually exclusive exons are defined by two cancer specific splice site pairs, alternative to one or two normal specific splice site pairs.

To detect cancer (or normal) specific cassette exons, we selected those genes characterized by the simultaneous occurrence of at least one cancer specific splice site and at least one normal specific splice site defining overlapping introns (Table 1). The presence of a tumoral (or normal) cassette exon or mutually exclusive exons was defined by two cancer specific splice site pairs, alternative to one or two normal specific splice site pairs (or viceversa) (Figure 2).

We identified a total of 46 genes (for two of them, HLA-B and HLA-C, predictions were related to more than one anatomical group) with 88 condition-specific cassette or mutually exclusive exons, specifically 34 tumor-specific exons and 54 normal-specific exons which are spliced out in the tumoral isoforms. Interestingly, only one gene (SLC25A3) revealed mutually exclusive exons. For any of these cassette exons a differential expression in the normal and cancer conditions was previously reported.

We have investigated specific functional feature enrichments in these 46 genes by using DAVID [31] and Ingenuity Pathway Analysis [29]. Results are reported in Additional Files 6 and 7. Interestingly, the two top networks associated to this gene set concern cell function and maintenance and cell cycle. Also, an over-representation of cancer associated genes (23/46) can be detected. Concerning structural properties of the protein sequences encoded by cassette exons, a remarkable domain enrichment can be observed as 36/46 cassette exons overlapped annotated domains (78.3%) in contrast to the 54.4% (111,456/204,934) generally observed for protein coding internal exons in human genes.

Table 2 lists all genes for which we identified tumoral- or normal-specific cassette exons, including mutually exclusive exons (see Additional File 8 for a full list of the cassette exons).

**Table 2:** Genes containing normal (N) or tumor (T)-specific cassette exons predicted *in silico*

Gene	Tissue(s)	Cassette Exon(s)	Description
ACTN1	CNS	N	actinin, alpha 1
ALDOA	TES	N	aldolase A, fructose-bisphosphate
ATP6V0A1	CNS	N	ATPase, H+ transporting, lysosomal V0 subunit a1
CS	CNS	T	citrate synthase
CTNNA1	CNS	N	catenin (cadherin-associated protein), alpha 1, 102kDa
DCTN1	CNS	N/T	dynactin 1 (p150, glued homolog, Drosophila)
EEF1D	EYE	T	eukaryotic translation elongation factor 1 delta (guanine nucleotide exchange protein)
EWSR1	CNS	N	Ewing sarcoma breakpoint region 1
FAM104A	TES	N	family with sequence similarity 104, member A
FAM49B	CNS	N	family with sequence similarity 49, member B
GSN	KID	T	gelsolin (amyloidosis, Finnish type)
HDLBP	CNS	T	high density lipoprotein binding protein
HLA-B	RES, LYM, MSK	N/T	major histocompatibility complex, class I, B
HLA-C	LYM, MSK, DER	N/T	major histocompatibility complex, class I, C
HLA-DRB1	CNS	T	major histocompatibility complex, class II, DR beta 1
HSPA8	DER	T	heat shock 70 kDa protein 8
IARS	CNS	N	isoleucyl-tRNA synthetase
IDH3A	LYM	N	isocitrate dehydrogenase 3 (NAD+) alpha
ITIH4	LIV	N	inter-alpha (globulin) inhibitor H4
LDHA	DER	N	lactate dehydrogenase A
LMNA	DER	T	lamin A/C
METT10D	CNS	T	methyltransferase 10 domain containing protein
MYL6	PLA	N	myosin, light chain 6, alkali, smooth muscle and non-muscle
MVK	CNS	N	mevalonate kinase
NAP1L1	MSK	T	nucleosome assembly protein 1-like 1
NDRG4	CNS	N	N-myc downstream regulated gene family member 4
NRSN2	CNS	N	neurensin 2
PCNP	CNS	N	PEST proteolytic signal containing nuclear protein
PKM2	TES	N	pyruvate kinase, muscle
PLEKHB1	EYE	N	pleckstrin homology domain containing, family B member 1
POMT1	TES	T	protein-O-mannosyltransferase 1
PRKCZ	PLA	T	protein kinase C, zeta
RAN	RES	T	RAN, member RAS oncogene family
RPH3A	CNS	N	rabphilin 3A homolog (mouse)
RPS24	MSK	N	ribosomal protein S24
SELENBP1	CNS	N	selenium binding protein 1
SLC25A3	MSK	N/T	solute carrier family 25 (mitochondrial phosphate carrier), member 3
STRADA	CNS	N	STE20-related kinase adaptor alpha
TMEM87A	CNS	N	transmembrane protein 87A
TPD52L2	MSK	N	tumor protein D52-like 2
TPM3	MSK	N	tropomyosin 3
UQCC	CNS	N	ubiquinol-cytochrome c reductase complex chaperone
WARS	LYM	T	tryptophanyl-tRNA synthetase
YPEL5	CNS	T	yippee-like 5 (Drosophila)
ZFAND6	TES	N	zinc finger, AN1-type domain 6
ZNF655	CNS	T	zinc finger protein 655

### Experimental validation of specific cassette exons by RT-qPCR

To experimentally validate our *in silico *predictions of the condition-specific cassette exons, we selected 7 normal-predicted cassette exons (detected in ATP6v0A1, STRADA, PCNP, TPM3 and TPD52L2 genes), 4 tumoral-predicted cassette exons (detected in CS, METT10 D and NAP1L1 genes) and 1 pair of mutually exclusive-predicted exons (detected in SLC25A3 gene) (Table 3).

**Table 3:** Predicted normal (N) and tumoral (T) cassette exons analyzed by RT-qPCR.

Anatomical group	**Gene**^**1**^	Exon(s) labels	Exon(s) absolute coordinates	Exon(s) length (bp)	Predicted expression specificity
CNS	ATP6V0A1 [38]	A	Chr17:37919833-37920004	172	N

CNS	CS [39]	B	Chr12:54971682-54971844	163	T

CNS	METT10 D [40]	C	Chr17:2325285-2325343	59	TT

CNS	PCNP [41]	D	Chr3:102786971-102787045	75 + 56	NN
		E	Chr3:102791749-102791804		

CNS	STRADA [42]	F	Chr17:59157730-59157787	58 + 29	N
		G	Chr17:59154390-59154418		

MSK	NAP1L1 [43]	H	Chr12:74748956-74749041	86 + 103	TT
		I	Chr12:74747418-74747520		

MSK	SLC25A3 [44]	J	Chr12:97513636-97513757	122	TT

MSK	SLC25A3	K	Chr12:97513342-97513466	125	N

MSK	TPD52L2 [45]	L	Chr20:61977613-61977672	60	NN

MSK	TPM3 [46]	M	Chr1:152408405-152408483	79	NN

^1 ^In the brackets are gene specific bibliographic references.

The anatomical group, the gene name, the exon label, genomic coordinates (NCBI36/hg18), exon length and predicted expression specificity are reported.

**Figure 3:**
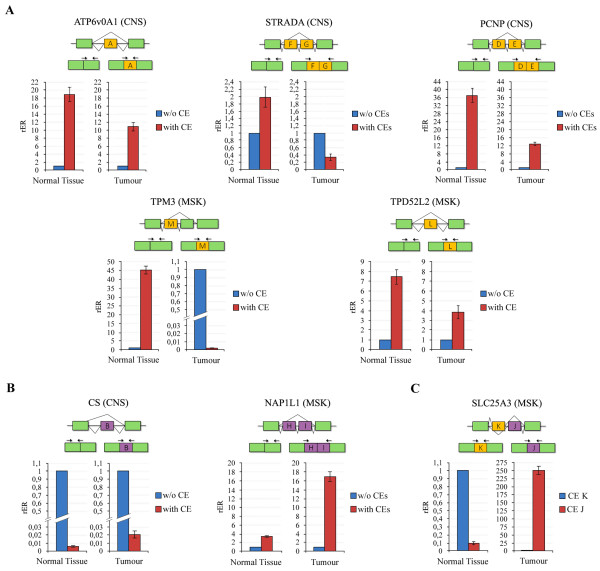
**Expression analyses of some normal/tumoral predicted cassette exons**. Experimental validation of normal **(A)**, and tumoral **(B) **predicted cassette exons (CE) and a pair of mutually exclusive CEs **(C) **by RT-qPCR. w/o CE = transcripts lacking CE; with CE = transcripts containing CE. For each condition, results are expressed as relative expression ratio of transcripts containing CE respect to transcripts lacking CE, used as calibrator. For each gene, a schematic representation of alternative transcripts and primer pairs used are reported.

We performed RT-qPCR assays, using total RNA derived from the same type of biological source (normal and tumoral) in which they were predicted. Notably, we compared the presence of the specific cassette exons in RNA from normal brain and neuroblastoma (for ATP6v0A1, STRADA, PCNP and CS) and from skeletal muscle and rhabdomyosarcoma (for TPM3, TPD52L2, NAP1L1, METT10 D and SLC25A3). Primers were designed to specifically amplify transcripts without or with cassette exons (w/o CE and with CE) (Figure 3). For each gene, we evaluated the relative expression ratio (rER) between transcripts containing cassette exons and transcripts without cassette exons in both normal and tumoral conditions of the anatomical group. We found that all computational predictions, but one, were validated in the *in vivo *analysis. For ATP6v0A1, STRADA, PCNP, TPM3 and TPD52L2 genes, the relative quantification analysis confirmed the presence of the normal-predicted specific cassette exons (Figure 3A).

For ATP6v0A1 gene, we observed that the normal predicted exon A, which is known to be included in the three transcript variants of this gene, is present in both normal and tumoral conditions, although it was prevalent in the normal brain sample.

For STRADA gene, multiple transcripts were described, some of them missing exon F and/or G, which encode for a portion of a kinase domain. We observed that, in normal brain, transcripts containing both F and G cassette exons are ~2-fold more abundant than the others and they are ~3-fold less expressed respect to transcripts lacking them in neuroblastoma. Therefore, F and G cassette exons showed a normal condition association, as predicted.

For PCNP gene, our computational analysis predicted two adjacent cassette exons, D and E, as normal-specific. We found that transcripts containing both these exons are more abundant than those lacking them in both normal and tumoral conditions, although they are ~3-fold less expressed in neuroblastoma respect to normal brain. Therefore, we classified exons D and E as normal cassette exons.

For TPM3 gene, multiple transcripts were reported, but only isoform 1 (NM_152263.2) contains cassette exon M. We found that this transcript is expressed at very low level respect to transcripts lacking exon M in the tumoral condition (more than 20,000 fold less), validating exon M as normal specific cassette exon.

For TPD52L2 gene, multiple transcripts were described, some of which lack exon L that we analyzed. We confirmed that exon L is normal-associated, as predicted. Indeed, we found that exon L containing transcripts are more expressed in both conditions respect to those lacking it, but they show a reduction (~2-fold) of their expression in rhabdomyosarcoma.

Figure 3B shows results for tumoral-specific cassette exons validation for CS and NAP1L1 genes.

For CS gene, our computational analysis predicted exon B as tumor-specific. We found that transcripts containing this exon are very low expressed, both in absolute terms and respect to transcripts missing it. Nevertheless, we observed that in neuroblastoma there is a ~3-fold increase of exon B presence respect to normal brain, allowing us to validate exon B as tumor-associated.

For NAP1L1 gene, two adjacent cassette exons, H and I, were predicted as tumoral specific. We observed that NAP1L1 transcripts including these cassette exons are expressed in both normal and tumoral conditions, but they are much more expressed in rhabdomyosarcoma respect to normal skeletal muscle tissue (about 5-fold).

Figure 3C shows the analysis of a pair of mutually exclusive cassette exons, K and J, for SLC25A3 gene. We confirmed the *in silico *predictions, as normal-predicted exon K (considered as calibrator) is more abundant than exon J (~10-fold) in normal skeletal muscle tissue and less expressed (~250-fold) in rhabdomyosarcoma.

The RT-qPCR assays did not validate the tumor-specific cassette exon predicted for METT10D in MSK, as it resulted as normal-specific (data not shown). This could be probably due to the heterogeneity of the ESTs source used for the computational analysis, likely different from the one used for the experimental assay.

**Figure 4:**
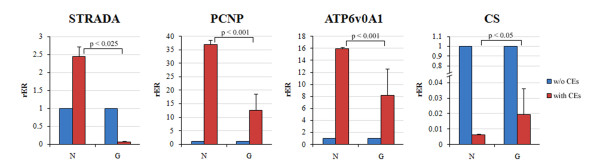
**Expression analyses of some CNS predicted cassette exons in gliobastoma samples**. Experimental validation of nervous system specific predicted cassette exons (CEs) of STRADA, PCNP, ATP6v0A1 and CS genes by RT-qPCR in normal brains and in 8 patients affected by glioblastoma. Statistical analysis was performed using an unpaired one-tail T-test. w/o CE = transcripts lacking CE; with CE = transcripts containing CE. For each condition, results are expressed as relative expression ratio of transcripts containing CE respect to transcripts lacking CE, used as calibrator.

The validation of the cassette exons specific for the nervous system, detected for ATP6v0A1, STRADA, PCNP and CS genes, prompted us to analyze their expression in patients affected by glioblastoma, the most frequent form of primary intracranial malignancy. Results are reported in Figure 4. We found that, respect to normal samples, STRADA and PCNP transcripts containing the predicted normal specific exons are less expressed in all tumoral samples respect to those lacking them. In particular, for STRADA gene we observed a switch between the two classes of transcripts, with a strong reduction of transcripts with the two CEs encoding the kinase domain of STRADA protein. For ATP6v0A1 gene, transcripts containing the predicted normal cassette exon are more expressed respect to those lacking it in tumoral samples, although their expression is reduced respect to the normal samples. Finally, for CS gene, although transcripts containing the tumoral predicted cassette exon are less expressed respect to those lacking it in all normal and tumoral samples, we found that these transcripts are more expressed in tumoral samples respect to normal brain samples.

## Discussion

Alternative splicing is widely recognized as a post-transcriptional regulatory mechanism which plays an important role in modulating gene expression in different tissues and developmental stages. Moreover, some alternatively spliced isoforms are associated with different diseases including cancer [5,23,24,32]. Actually, mutations at the exon-intron junctions or affecting exonic or intronic splicing regulatory elements can contribute to the development of neoplastic states.

Histological information deriving from EST libraries annotation can be very useful in defining the expression profile of tumoral cells. The presence of a high number of tumoral ESTs and the absence of normal ESTs (or viceversa) mapping on a precise genomic region could mean that, in a cell, the presence/absence of a gene product may be cause or consequence of the tumoral pathology. The observation that the same gene may express many structurally and functionally different products, not only changed radically the traditional gene definition [33] but also extended the perspective of the analysis of differential expression in the normal and tumoral conditions beyond the gene level, taking into account alternative transcripts and proteins.

In this work, we have focused our attention on the study of alternative splicing aimed to the identification of cancer specific isoforms. Our method involves the characterization of splice sites with information deriving from EST libraries despite some intrinsic limitations of such data (34). Several studies of differential expression between the normal and tumoral conditions have been previously reported [23,24,35] through *in silico *analyses. Some predictions were also experimentally validated and collected in specialized databases, like ASAPII [13]. The method we developed for the classification of normal and tumoral introns differs from that used in ASAPII for two main reasons: 1) the statistics used; 2) the logic adopted for the definition of cancer specificity. Indeed, a serious limitation of the ASAPII database is that cancer specificity is detached from the tissue information as introns are classified cancer-specific regardless of the source tissue. In this way, as the Authors also report, some information can be lost or hidden by the general expression pattern of the gene, which may be tissue-specific. For example, in our analyses, for the KRT7 gene, the same splice site can be classified as normal- or cancer-specific depending on the tissue type considered (see Additional File 9). Such type of introns remain unclassified in the ASAPII database because it merges together ESTs from different tissues thus causing the flattening of the signal.

Another difference is represented by the fact that we define the splice site specificity in an absolute manner while in ASAPII database the tissue or histological specificity of each splice site is defined in comparison to another splice site. For the Tryptophanyl-tRNA synthetase gene (WARS), for example, the same splice site has been classified tumoral or normal depending on the other splice site with which it was compared to. In this way each splice site can not be univocally classified.

We approached the study of alternative splicing and its association to cancer, focusing, in the first part of the work, on the gene differential expression in normal and tumoral conditions among different tissues, in order to exclude from our candidate cancer specific splicing events, splice sites belonging to genes expressed in only one histological condition. We performed the classification analysis at intron level. The observation that CNS is the tissue with the highest number of tumor-specific splicing could imply some biological meaning or it could simply reflect the larger number of available normal and tumoral ESTs for the statistical validation.

Moreover, we specifically focused our attention on cassette exons to increase the reliability of statistical assessments, because of the necessity of a simultaneous occurrence of statistically validated tumor and normal-specific splicing sites, and to provide a collection of tumor biomarkers easily assayable and potentially suitable to be tested as therapeutic targets (e.g. using specific antibodies carrying cytotoxic agents).

Given the genome-wide approach (more than 18,000 genes analyzed), the number of significant results, a total of 88 cases, may not seem very high, but it must be recalled that we have used very stringent criteria which require simultaneous EST support for at least three splicing sites in both the normal and the tumoral status (see the data-flux flowchart in the Additional File 10). Furthermore, it should be considered that differential expression was based on EST counts from pooling normal and tumor libraries, thus loosing the replicate information. Forthcoming efforts using the huge amount of data provided by next-generation sequencing could suitably address this issue.

Our methodology has not identified all genes known to be characterized by the presence of tumor specific isoforms. For some genes like MDM2 and MDM4 [5], this was due to the insufficient number of available ESTs, either tumoral or normal, to define intron histological specificity. For CD44 [23] or TP53 [36] genes, we found cancer-specific splice sites in many tissues, but the number of normal ESTs was not sufficient to find significant normal-specific introns. Beside the small number of ESTs supporting introns prediction, another problem encountered has been the lack of ESTs annotation. The absence of information concerning tissue or histology associated to libraries led us to the exclusion of many EST sequences from the classification analysis. It is thus essential to reaffirm the importance of a good sequence annotation.

Finally, in order to provide a more accurate and quantitative estimate of the expression specificity of the predicted cassette exons, we carried out RT-qPCR assays. We found that all predicted normal or tumoral specific transcripts, but one, were confirmed *in vivo *in the same type of biological source (normal and tumoral) in which they were predicted. Of particular interest was the specific expression of ATP6v0A1, STRADA, PCNP and CS transcript variants, analyzed in patients affected by glioblastoma, which confirmed all the predicted cassette exons, thus demonstrating the reliability of our computational analysis. In particular, the cassette exons we identified in the STRADA gene represent a very promising biomarker as their significant differential expression in the normal and cancer conditions is homogeneously conserved in all samples analyzed.

## Conclusion

We present a novel computational strategy for *in silico *identification of tumor-associated transcript variants to be used as cancer molecular biomarkers, that was supported by the experimental validation which demonstrated that cassette exons are differently associated to normal/tumoral condition. In particular, we assessed the biomarker suitability of some cassette exons, found differentially expressed in the nervous system, in patients affected by glioblastoma.

This methodology can be easily adapted to manage exon array hybridization data [4] and next-generation sequencing data [37], thus opening unprecedented opportunities for a thorough investigation of the expression pattern in human and other organisms.

## Abbreviations

AS: alternative splicing; ESTs: Expressed Sequence Tag sequences; RT-qPCR: Reverse Transcriptase-quantitative Polymerase Chain Reaction.

## Competing interests

The authors declare that they have no competing interests.

## Authors' contributions

AV and MM performed experimental validations; AA and IB performed computational analyses; FM supervised the computational analyses; GM and VD provided gliomablastoma samples; AT and ES participated in the discussion and final manuscript preparation; AMD supervised experimental validations and wrote the manuscript; GP designed and coordinated the study and wrote the manuscript. All authors read and approved the final version of this manuscript.

## Supplementary Material

Additional file 1 **Primers used in RT-qPCR validations**. This table reports the sequences of all primers used in the validation experiments and the relative amplicon sizes. Click here for file

Additional file 2 **List of 1375 genes exclusively expressed in the tumor status and tissue**. For each gene this table reports the tissue type (see the legend of Fig. 1), the number of ESTs from tumor libraries and their total size, the p-value calculated as described in the Method section. A total of 469 genes result statistically significant also after the Bonferroni correction (*) at 0.05 confidence level. Click here for file

Additional file 3 **Top 10 represented GO terms among genes over-represented in tumor (T/TT) and normal (N/NN) tissues**. For each GO term this table reports the GO identifier (GO_Id), the number of genes, the P value (P-val) and the description (GO_Description). Only Gene Ontology terms assigned to more than 50 human genes were considered. Click here for file

Additional file 4 **Genes and splicing sites showing a normal or tumor specific expression**. For each tissue this table reports the number of genes and splicing sites with a labeled expression pattern as described in the text (total numbers after the Bonferroni correction at the 0.05 confidence level are in the brackets) Click here for file

Additional file 5 **Heterogeneous classification of the first intron of the PPP2R1A gene (intron #4 following ASPidDB nomenclature located at chr19:57385240-57397008)**. Expression pattern of intron #4 of the PPP2R1A gene where is shown the number of supporting ESTs from normal and cancer tissues, the statistical significance and the classification. This intron is exclusively expressed in tumor bone marrow (BMA) and in normal endocrine system (END) (see red arrows). Click here for file

Additional file 6 **Functional analysis of 46 genes characterized by a normal- or tumoral- specific cassette exon**. The file contains enriched biological terms identified with DAVID (**D**atabase for **A**nnotation, **V**isualization and **I**ntegrated **D**iscovery) v6.7 (page 1). Most significant terms include "acetylation", "phosphoprotein" and "alternative splicing". An Ingenuity Pathway Analysis, which delivers an assessment of the signalling and metabolic pathways, molecular networks, and biological processes that are most significantly perturbed in a dataset of interest, is also included (pages 2-8). The "Top Networks" section contains the four networks, with a statistically significant score, that could be detected from the input dataset. The network with the highest score (score = 42) is significantly enriched in the following functions: *Cell-mediated Immune Response, Cellular Function and Maintenance, Hematological System Development and Function*. The report also includes (pages 7 and 8) a graphical representation of two top networks which comprise genes belonging to the network and the relationships among them. Other key components of the IPA Core Analysis, shown in the report, are: "Diseases and Disorders", "Molecular and Cellular Functions" and "Physiological System Development and Function". The number of molecules (i.e. genes) belonging to the different classes and associated p-values are reported. Click here for file

Additional file 7 **IPA analysis data**. A gene list for each of the four top functional networks is reported in the "Top_network" sheet. The "CE_proteindomains" sheet lists the protein domains encoded by the condition-specific (normal or tumoral) cassette exons. Click here for file

Additionale file 8 **List of predicted normal (A) and tumor (B) -specific cassette exons**. The anatomical group, the gene name, the genomic coordinates (NCBI36/hg18), and the unique transcript ID following Riva and Pesole (2009) are reported. Click here for file

Additional file 9 **Expression pattern of intron #12 of KRT7 gene**. ASPicDB table relative to the expression pattern of intron #12 of the KRT7 gene (following ASPicDB nomenclature, located at chr12:50917621-50918730) showing the number of supporting ESTs from normal and cancer tissues, the statistical significance and the classification. Click here for file

Additional file 10 **Data flux flowchart**. The flowchart reports the number of genes at each step in the detection of normal or cancer-specific cassette exons. Click here for file
