# Supplementary material for: Identification of tumor-associated cassette exons in human cancer through EST-based computational prediction and experimental validation
Source: Mol Cancer. 2010 Sep 2;9:230. doi: 10.1186/1476-4598-9-230 (PMC2941758; doi:10.1186/1476-4598-9-230)
Supplement: Additional file 1 — Primers used in RT-qPCR validations. This table reports the sequences of all primers used in the validation experiments and the relative amplicon sizes. [file 1476-4598-9-230-S1.DOC]

| **Primer Name** | **Sequence 5'→3'** | **Amplicon Size** |
| --- | --- | --- |
| ATP6v0A1 ex45-52 For | GCTCATGCGCGGTTGAGTTCC | 105bp |
| ATP6v0A1 ex52 Rev | CTCACTCTTCAAACTTCCCTTCC |
| ATP6v0A1 ex45-47 For | CGCTCATGCGCAGCTGTCTGA | 194bp |
| ATP6v0A1 ex47-52 Rev | GGAACTCAACCCAGTGTAAGCG |
| STRADA ex30-22 For | GCGAATCAGGACCAATGATGC | 107bp |
| STRADA ex22 Rev | CAGTGAGCAGCTCGTAACACC |
| STRADA ex30-28 For | CGAATCAGGCGGTGGGTCTC | 104bp |
| STRADA ex26-22 Rev | TCATTGGTTTTTCTCCGAGTGTC |
| PCNP ex12-22 For | GGATCAAGTGGATACACCAACA | 161bp |
| PCNP ex22 Rev | CCACACCCCAATTTCAAAACATC |
| PCNP ex12-17 For | GGATCAAGTAAGCCTAAAGAAAC | 148bp |
| PCNP ex21-22 Rev | GTGTATCCCTTCCAATATTCTTC |
| TPM3 ex17-2 For | ATGACCTGGAAGATAAACTGAAATG | 86bp |
| TPM3 ex2 Rev | ATTCAGGTCAAGCAGGGTCTGG |
| TPM3 ex17-15 For | GACCTGGAAGATGAGCTCTATG | 102bp |
| TPM3 ex15-13 Rev | CGGTGATAATTATATAGAGGTCATG |
| TPD52L2 ex10-16 For | CTAGCGCCTACAAGAAGACTCA | 88bp |
| TPD52L2 ex16 Rev | CTGATGGCAGAGCCCACTGT |
| TPD52L2 ex10-14 For | CTAGCGCCTATGTGAAAACTTC | 74bp |
| TPD52L2 ex14-16 Rev | CTTGTAGAGGTCTGACTGGGT |
| CS ex40 For | CCGCCTCCTTTCAACCTTGTC | 169bp |
| CS ex40-28 Rev | CATTCTTGGTTCCCAAGAGC |
| CS ex39-32 For | ACCAAGGGAGCTGACCTATTG | 174bp |
| CS ex32-28 Rev | CATTCTCTGAATCATCCCTGAAC |
| METT10D ex24-21 For | GACATAGGCACGGGGGCATC | 71bp |
| METT10D ex21 Rev | GCGAGGAAATACCAGCCATTC |
| METT10D ex24-22 For | GACATAGGTATCCAAGGACGTG | 73bp |
| METT10D ex22-21 Rev | CCCGTGCCATTTTTGCCCGA |
| NAP1L1 ex45-37 For | CATTGACAACCTGCCTAGGGT | 145bp |
| NAP1L1 ex37 Rev | AAATAGAGGCTGATAGAGAACAG |
| NAP1L1 ex45-42 For | TGACAACAAAGAACAGTCTGAAC | 203bp |
| NAP1L1 ex40-37 Rev | TAGGCAGGCTTTCAATGTATCC |
| SLC25A3n ex10-15 For | GCCGTGGAAGAGCAGTATAGC | 145bp |
| SLC25A3n ex15-20 Rev | GGGGGTCCACCTGCATTCTG |
| SLC25A3t ex10-17 For | GCCGTGGAAGAGTACAGTTGTG | 142bp |
| SLC25A3t ex17-20 Rev | GGGGGTCCACCTGCATACGG |
