# Supplementary material for: Identification of tumor-associated cassette exons in human cancer through EST-based computational prediction and experimental validation
Source: Mol Cancer. 2010 Sep 2;9:230. doi: 10.1186/1476-4598-9-230 (PMC2941758; doi:10.1186/1476-4598-9-230)
Supplement: Additional file 2 — List of 1375 genes exclusively expressed in the tumor status and tissue. For each gene this table reports the tissue type (see the legend of Fig. 1), the number of ESTs from tumor libraries and their total size, the p-value calculated as described in the Method section. A total of 469 genes result statistically significant also after the Bonferroni correction (*) at 0.05 confidence level. [file 1476-4598-9-230-S2.DOC]

| **Tissue** | **Gene** | **EST T** | **Tot EST T** | **p_value** |  |
| --- | --- | --- | --- | --- | --- |
| BMA | AARS | 22 | 31251 | 1,44E-05 | (*) |
| BMA | ABI1 | 13 | 31251 | 1,38E-03 |  |
| BMA | ACSM3 | 21 | 31251 | 2,39E-05 | (*) |
| BMA | AHCY | 22 | 31251 | 1,44E-05 | (*) |
| BMA | ALDH1A1 | 14 | 31251 | 8,30E-04 |  |
| BMA | AMZ2 | 10 | 31251 | 6,30E-03 |  |
| BMA | ANAPC13 | 12 | 31251 | 2,29E-03 |  |
| BMA | ANXA3 | 11 | 31251 | 3,80E-03 |  |
| BMA | ANXA7 | 10 | 31251 | 6,30E-03 |  |
| BMA | APLP2 | 26 | 31251 | 1,89E-06 | (*) |
| BMA | ARF4 | 18 | 31251 | 1,09E-04 |  |
| BMA | ATP5A1 | 18 | 31251 | 1,09E-04 |  |
| BMA | ATP5F1 | 24 | 31251 | 5,22E-06 | (*) |
| BMA | ATP5G3 | 13 | 31251 | 1,38E-03 |  |
| BMA | C14ORF147 | 18 | 31251 | 1,09E-04 |  |
| BMA | CALU | 13 | 31251 | 1,38E-03 |  |
| BMA | CCT8 | 13 | 31251 | 1,38E-03 |  |
| BMA | CNIH | 29 | 31251 | 4,14E-07 | (*) |
| BMA | COASY | 12 | 31251 | 2,29E-03 |  |
| BMA | DAZAP2 | 10 | 31251 | 6,30E-03 |  |
| BMA | EEF2 | 24 | 31251 | 5,22E-06 | (*) |
| BMA | EIF3I | 43 | 31251 | 2,91E-10 | (*) |
| BMA | EIF4E | 42 | 31251 | 5,60E-10 | (*) |
| BMA | EIF5B | 10 | 31251 | 6,30E-03 |  |
| BMA | FUBP1 | 10 | 31251 | 6,30E-03 |  |
| BMA | GDI2 | 25 | 31251 | 3,14E-06 | (*) |
| BMA | GPI | 110 | 31251 | 0,00E+00 | (*) |
| BMA | HADHA | 12 | 31251 | 2,29E-03 |  |
| BMA | HAX1 | 10 | 31251 | 6,30E-03 |  |
| BMA | HDAC2 | 16 | 31251 | 3,01E-04 |  |
| BMA | HELLS | 24 | 31251 | 5,22E-06 | (*) |
| BMA | HIGD1A | 150 | 31251 | 1,93E-11 | (*) |
| BMA | HMGA1 | 11 | 31251 | 3,80E-03 |  |
| BMA | HMGB2 | 11 | 31251 | 3,80E-03 |  |
| BMA | HNRNPF | 16 | 31251 | 3,01E-04 |  |
| BMA | HNRNPH1 | 14 | 31251 | 8,30E-04 |  |
| BMA | HNRNPK | 17 | 31251 | 1,81E-04 |  |
| BMA | HSP90AB1 | 69 | 31251 | 0,00E+00 | (*) |
| BMA | HYOU1 | 21 | 31251 | 2,39E-05 | (*) |
| BMA | IDI1 | 18 | 31251 | 1,09E-04 |  |
| BMA | IGJ | 57 | 31251 | 0,00E+00 | (*) |
| BMA | KIAA0090 | 15 | 31251 | 5,00E-04 |  |
| BMA | KIF2C | 11 | 31251 | 3,80E-03 |  |
| BMA | KPNB1 | 17 | 31251 | 1,81E-04 |  |
| BMA | LILRA1 | 41 | 31251 | 9,17E-10 | (*) |
| BMA | MAPKSP1 | 11 | 31251 | 3,80E-03 |  |
| BMA | MKI67IP | 16 | 31251 | 3,01E-04 |  |
| BMA | MLLT11 | 57 | 31251 | 0,00E+00 | (*) |
| BMA | NDUFA9 | 13 | 31251 | 1,38E-03 |  |
| BMA | NDUFV1 | 10 | 31251 | 6,30E-03 |  |
| BMA | NET1 | 16 | 31251 | 3,01E-04 |  |
| BMA | NIP7 | 10 | 31251 | 6,30E-03 |  |
| BMA | NOP56 | 15 | 31251 | 5,00E-04 |  |
| BMA | NOSTRIN | 12 | 31251 | 2,29E-03 |  |
| BMA | OAT | 10 | 31251 | 6,30E-03 |  |
| BMA | OLA1 | 10 | 31251 | 6,30E-03 |  |
| BMA | PDCD6IP | 11 | 31251 | 3,80E-03 |  |
| BMA | PL-5283 | 23 | 31251 | 8,67E-06 | (*) |
| BMA | PPP2R1A | 10 | 31251 | 6,30E-03 |  |
| BMA | PRAME | 26 | 31251 | 1,89E-06 | (*) |
| BMA | PSMA4 | 45 | 31251 | 7,72E-11 | (*) |
| BMA | PTGES3 | 10 | 31251 | 6,30E-03 |  |
| BMA | PXMP3 | 13 | 31251 | 1,38E-03 |  |
| BMA | RBBP4 | 13 | 31251 | 1,38E-03 |  |
| BMA | RDH11 | 15 | 31251 | 5,00E-04 |  |
| BMA | RFC4 | 16 | 31251 | 3,01E-04 |  |
| BMA | RPN1 | 10 | 31251 | 6,30E-03 |  |
| BMA | RPS23 | 26 | 31251 | 1,89E-06 | (*) |
| BMA | RPS9 | 14 | 31251 | 8,30E-04 |  |
| BMA | RRM2 | 12 | 31251 | 2,29E-03 |  |
| BMA | SELT | 35 | 31251 | 1,96E-08 | (*) |
| BMA | SERPINH1 | 19 | 31251 | 6,58E-05 |  |
| BMA | SH3BGRL | 15 | 31251 | 5,00E-04 |  |
| BMA | SLC2A3 | 10 | 31251 | 6,30E-03 |  |
| BMA | SND1 | 10 | 31251 | 6,30E-03 |  |
| BMA | SNRNP27 | 10 | 31251 | 6,30E-03 |  |
| BMA | SNW1 | 13 | 31251 | 1,38E-03 |  |
| BMA | TBPL1 | 14 | 31251 | 8,30E-04 |  |
| BMA | TFEB | 17 | 31251 | 1,81E-04 |  |
| BMA | TGM2 | 10 | 31251 | 6,30E-03 |  |
| BMA | TKT | 13 | 31251 | 1,38E-03 |  |
| BMA | TM4SF1 | 12 | 31251 | 2,29E-03 |  |
| BMA | TMEM126B | 10 | 31251 | 6,30E-03 |  |
| BMA | TPMT | 11 | 31251 | 3,80E-03 |  |
| BMA | TRAP1 | 10 | 31251 | 6,30E-03 |  |
| BMA | TUBB4 | 13 | 31251 | 1,38E-03 |  |
| BMA | UBE2V2 | 142 | 31251 | 0,00E+00 | (*) |
| BMA | VBP1 | 16 | 31251 | 3,01E-04 |  |
| BMA | VRK1 | 16 | 31251 | 3,01E-04 |  |
| BMA | YWHAE | 10 | 31251 | 6,30E-03 |  |
| BMA | ZNF207 | 28 | 31251 | 6,87E-07 | (*) |
| BRE | ABCC11 | 16 | 141303 | 3,59E-03 |  |
| BRE | ACO2 | 19 | 141303 | 1,25E-03 |  |
| BRE | AIF1L | 23 | 141303 | 3,06E-04 |  |
| BRE | ALDH3B2 | 26 | 141303 | 1,06E-04 |  |
| BRE | ANP32B | 16 | 141303 | 3,59E-03 |  |
| BRE | ARF5 | 14 | 141303 | 7,25E-03 |  |
| BRE | ARPC1A | 22 | 141303 | 4,34E-04 |  |
| BRE | ASAH1 | 39 | 141303 | 1,10E-06 | (*) |
| BRE | ASCC2 | 17 | 141303 | 2,52E-03 |  |
| BRE | ATP6V1A | 17 | 141303 | 2,52E-03 |  |
| BRE | BAT2 | 18 | 141303 | 1,78E-03 |  |
| BRE | BLVRB | 23 | 141303 | 3,06E-04 |  |
| BRE | BSDC1 | 20 | 141303 | 8,78E-04 |  |
| BRE | C17ORF45 | 34 | 141303 | 6,37E-06 | (*) |
| BRE | C1QBP | 15 | 141303 | 5,10E-03 |  |
| BRE | C20ORF149 | 28 | 141303 | 5,26E-05 |  |
| BRE | C20ORF43 | 21 | 141303 | 6,18E-04 |  |
| BRE | C2ORF28 | 35 | 141303 | 4,48E-06 | (*) |
| BRE | CCNB2 | 15 | 141303 | 5,10E-03 |  |
| BRE | CD276 | 19 | 141303 | 1,25E-03 |  |
| BRE | CDC37 | 19 | 141303 | 1,25E-03 |  |
| BRE | CDK4 | 15 | 141303 | 5,10E-03 |  |
| BRE | CDK5RAP3 | 28 | 141303 | 5,26E-05 |  |
| BRE | COPE | 21 | 141303 | 6,18E-04 |  |
| BRE | COPG | 29 | 141303 | 3,70E-05 |  |
| BRE | CORO1B | 19 | 141303 | 1,25E-03 |  |
| BRE | CYC1 | 20 | 141303 | 8,78E-04 |  |
| BRE | DCD | 21 | 141303 | 6,18E-04 |  |
| BRE | DDR1 | 26 | 141303 | 1,06E-04 |  |
| BRE | DDX21 | 14 | 141303 | 7,25E-03 |  |
| BRE | DDX50 | 14 | 141303 | 7,25E-03 |  |
| BRE | DOCK6 | 14 | 141303 | 7,25E-03 |  |
| BRE | DUS3L | 22 | 141303 | 4,34E-04 |  |
| BRE | DYNLL1 | 20 | 141303 | 8,78E-04 |  |
| BRE | EBNA1BP2 | 14 | 141303 | 7,25E-03 |  |
| BRE | EEF1A2 | 72 | 141303 | 2,89E-10 | (*) |
| BRE | EFEMP1 | 14 | 141303 | 7,25E-03 |  |
| BRE | EIF2A | 19 | 141303 | 1,25E-03 |  |
| BRE | EIF5A | 38 | 141303 | 1,56E-06 | (*) |
| BRE | ENG | 15 | 141303 | 5,10E-03 |  |
| BRE | ERBB2 | 35 | 141303 | 4,48E-06 | (*) |
| BRE | FAU | 23 | 141303 | 3,06E-04 |  |
| BRE | FKBP1A | 14 | 141303 | 7,25E-03 |  |
| BRE | FKBP8 | 24 | 141303 | 2,15E-04 |  |
| BRE | FMOD | 35 | 141303 | 4,48E-06 | (*) |
| BRE | FTSJ3 | 19 | 141303 | 1,25E-03 |  |
| BRE | FUCA1 | 18 | 141303 | 1,78E-03 |  |
| BRE | FUT8 | 26 | 141303 | 1,06E-04 |  |
| BRE | GFRA1 | 14 | 141303 | 7,25E-03 |  |
| BRE | GJA1 | 18 | 141303 | 1,78E-03 |  |
| BRE | GLTSCR2 | 20 | 141303 | 8,78E-04 |  |
| BRE | GMPPA | 15 | 141303 | 5,10E-03 |  |
| BRE | GPC3 | 16 | 141303 | 3,59E-03 |  |
| BRE | GPS1 | 18 | 141303 | 1,78E-03 |  |
| BRE | GSS | 16 | 141303 | 3,59E-03 |  |
| BRE | HK1 | 21 | 141303 | 6,18E-04 |  |
| BRE | HMGN2 | 29 | 141303 | 3,70E-05 |  |
| BRE | HNRNPA3 | 15 | 141303 | 5,10E-03 |  |
| BRE | HNRNPM | 15 | 141303 | 5,10E-03 |  |
| BRE | HSD17B10 | 16 | 141303 | 3,59E-03 |  |
| BRE | IDH3G | 63 | 141303 | 1,11E-10 | (*) |
| BRE | IDS | 19 | 141303 | 1,25E-03 |  |
| BRE | IFI6 | 20 | 141303 | 8,78E-04 |  |
| BRE | IK | 42 | 141303 | 3,81E-07 | (*) |
| BRE | IL17RC | 21 | 141303 | 6,18E-04 |  |
| BRE | IMMT | 17 | 141303 | 2,52E-03 |  |
| BRE | INTS7 | 18 | 141303 | 1,78E-03 |  |
| BRE | IRAK1 | 14 | 141303 | 7,25E-03 |  |
| BRE | LAPTM4A | 34 | 141303 | 6,37E-06 | (*) |
| BRE | LGALS3 | 29 | 141303 | 3,70E-05 |  |
| BRE | LRRC59 | 26 | 141303 | 1,06E-04 |  |
| BRE | LY6E | 14 | 141303 | 7,25E-03 |  |
| BRE | MAP2K2 | 19 | 141303 | 1,25E-03 |  |
| BRE | MCM6 | 19 | 141303 | 1,25E-03 |  |
| BRE | MEA1 | 14 | 141303 | 7,25E-03 |  |
| BRE | METTL13 | 14 | 141303 | 7,25E-03 |  |
| BRE | MLF2 | 21 | 141303 | 6,18E-04 |  |
| BRE | MMADHC | 21 | 141303 | 6,18E-04 |  |
| BRE | MPV17 | 15 | 141303 | 5,10E-03 |  |
| BRE | MRPL49 | 26 | 141303 | 1,06E-04 |  |
| BRE | MT2A | 24 | 141303 | 2,15E-04 |  |
| BRE | MTO1 | 88 | 141303 | 0,00E+00 | (*) |
| BRE | MYBL2 | 30 | 141303 | 2,60E-05 | (*) |
| BRE | MYO19 | 16 | 141303 | 3,59E-03 |  |
| BRE | NAMPT | 15 | 141303 | 5,10E-03 |  |
| BRE | NCOA3 | 15 | 141303 | 5,10E-03 |  |
| BRE | NME4 | 19 | 141303 | 1,25E-03 |  |
| BRE | NOC2L | 27 | 141303 | 7,48E-05 |  |
| BRE | NPR1 | 57 | 141303 | 2,15E-09 | (*) |
| BRE | NPY1R | 30 | 141303 | 2,60E-05 | (*) |
| BRE | OAS1 | 21 | 141303 | 6,18E-04 |  |
| BRE | PAK1 | 16 | 141303 | 3,59E-03 |  |
| BRE | PARK7 | 22 | 141303 | 4,34E-04 |  |
| BRE | PCNA | 18 | 141303 | 1,78E-03 |  |
| BRE | PDHA1 | 14 | 141303 | 7,25E-03 |  |
| BRE | PEBP1 | 21 | 141303 | 6,18E-04 |  |
| BRE | PIP5K1A | 18 | 141303 | 1,78E-03 |  |
| BRE | PLOD3 | 18 | 141303 | 1,78E-03 |  |
| BRE | PLP2 | 21 | 141303 | 6,18E-04 |  |
| BRE | PMM2 | 15 | 141303 | 5,10E-03 |  |
| BRE | POLDIP2 | 16 | 141303 | 3,59E-03 |  |
| BRE | POLR2E | 21 | 141303 | 6,18E-04 |  |
| BRE | POLR2J | 31 | 141303 | 1,83E-05 | (*) |
| BRE | POMGNT1 | 15 | 141303 | 5,10E-03 |  |
| BRE | PQBP1 | 16 | 141303 | 3,59E-03 |  |
| BRE | PRC1 | 18 | 141303 | 1,78E-03 |  |
| BRE | PRDX5 | 15 | 141303 | 5,10E-03 |  |
| BRE | PRLR | 35 | 141303 | 4,48E-06 | (*) |
| BRE | PSMB3 | 14 | 141303 | 7,25E-03 |  |
| BRE | PSMB7 | 18 | 141303 | 1,78E-03 |  |
| BRE | PSMB8 | 14 | 141303 | 7,25E-03 |  |
| BRE | PSMD4 | 16 | 141303 | 3,59E-03 |  |
| BRE | PSMD6 | 21 | 141303 | 6,18E-04 |  |
| BRE | PTTG1 | 19 | 141303 | 1,25E-03 |  |
| BRE | RARS | 17 | 141303 | 2,52E-03 |  |
| BRE | RBBP7 | 31 | 141303 | 1,83E-05 | (*) |
| BRE | RBM8A | 14 | 141303 | 7,25E-03 |  |
| BRE | RDBP | 14 | 141303 | 7,25E-03 |  |
| BRE | RNF4 | 18 | 141303 | 1,78E-03 |  |
| BRE | RPL10A | 21 | 141303 | 6,18E-04 |  |
| BRE | RPL12 | 70 | 141303 | 1,79E-10 | (*) |
| BRE | RPL14 | 259 | 141303 | 0,00E+00 | (*) |
| BRE | RPL19 | 46 | 141303 | 9,34E-08 | (*) |
| BRE | RPL36 | 22 | 141303 | 4,34E-04 |  |
| BRE | RPL7 | 54 | 141303 | 5,06E-09 | (*) |
| BRE | RPS13 | 50 | 141303 | 2,26E-08 | (*) |
| BRE | RPS14 | 58 | 141303 | 1,13E-09 | (*) |
| BRE | RPS15A | 31 | 141303 | 1,83E-05 | (*) |
| BRE | RPS18 | 65 | 141303 | 8,27E-11 | (*) |
| BRE | RPS2 | 42 | 141303 | 3,81E-07 | (*) |
| BRE | RPS26 | 17 | 141303 | 2,52E-03 |  |
| BRE | RPS27 | 21 | 141303 | 6,18E-04 |  |
| BRE | RPS9 | 45 | 141303 | 1,33E-07 | (*) |
| BRE | RRM2 | 15 | 141303 | 5,10E-03 |  |
| BRE | RRP1 | 14 | 141303 | 7,25E-03 |  |
| BRE | SAE1 | 16 | 141303 | 3,59E-03 |  |
| BRE | SAFB | 14 | 141303 | 7,25E-03 |  |
| BRE | SEC11C | 25 | 141303 | 1,51E-04 |  |
| BRE | SEMA3C | 22 | 141303 | 4,34E-04 |  |
| BRE | SEPHS1 | 15 | 141303 | 5,10E-03 |  |
| BRE | SEPW1 | 73 | 141303 | 5,31E-11 | (*) |
| BRE | SERBP1 | 14 | 141303 | 7,25E-03 |  |
| BRE | SERINC3 | 26 | 141303 | 1,06E-04 |  |
| BRE | SERPINA6 | 19 | 141303 | 1,25E-03 |  |
| BRE | SF1 | 14 | 141303 | 7,25E-03 |  |
| BRE | SFRS1 | 16 | 141303 | 3,59E-03 |  |
| BRE | SLC25A39 | 16 | 141303 | 3,59E-03 |  |
| BRE | SLC35A2 | 46 | 141303 | 9,34E-08 | (*) |
| BRE | SLC39A1 | 26 | 141303 | 1,06E-04 |  |
| BRE | SLC9A3R1 | 18 | 141303 | 1,78E-03 |  |
| BRE | SNRNP70 | 31 | 141303 | 1,83E-05 | (*) |
| BRE | SNRPB | 32 | 141303 | 1,29E-05 | (*) |
| BRE | SNRPB2 | 14 | 141303 | 7,25E-03 |  |
| BRE | SNRPD2 | 16 | 141303 | 3,59E-03 |  |
| BRE | SNRPF | 15 | 141303 | 5,10E-03 |  |
| BRE | SPAG5 | 17 | 141303 | 2,52E-03 |  |
| BRE | SRM | 15 | 141303 | 5,10E-03 |  |
| BRE | SSRP1 | 17 | 141303 | 2,52E-03 |  |
| BRE | STMN1 | 21 | 141303 | 6,18E-04 |  |
| BRE | STOML2 | 32 | 141303 | 1,29E-05 | (*) |
| BRE | SULF2 | 27 | 141303 | 7,48E-05 |  |
| BRE | SYAP1 | 16 | 141303 | 3,59E-03 |  |
| BRE | SYVN1 | 22 | 141303 | 4,34E-04 |  |
| BRE | TAF15 | 21 | 141303 | 6,18E-04 |  |
| BRE | TARS2 | 17 | 141303 | 2,52E-03 |  |
| BRE | TFRC | 19 | 141303 | 1,25E-03 |  |
| BRE | TH1L | 20 | 141303 | 8,78E-04 |  |
| BRE | TK1 | 40 | 141303 | 7,71E-07 | (*) |
| BRE | TOMM22 | 14 | 141303 | 7,25E-03 |  |
| BRE | TOP1MT | 14 | 141303 | 7,25E-03 |  |
| BRE | TOP3A | 14 | 141303 | 7,25E-03 |  |
| BRE | TPP1 | 20 | 141303 | 8,78E-04 |  |
| BRE | TPX2 | 15 | 141303 | 5,10E-03 |  |
| BRE | TRA2B | 17 | 141303 | 2,52E-03 |  |
| BRE | UBA52 | 28 | 141303 | 5,26E-05 |  |
| BRE | UBB | 15 | 141303 | 5,10E-03 |  |
| BRE | UBE2D3 | 20 | 141303 | 8,78E-04 |  |
| BRE | UBE2S | 14 | 141303 | 7,25E-03 |  |
| BRE | UQCRH | 20 | 141303 | 8,78E-04 |  |
| BRE | VPS72 | 24 | 141303 | 2,15E-04 |  |
| BRE | WDR46 | 14 | 141303 | 7,25E-03 |  |
| BRE | WISP2 | 20 | 141303 | 8,78E-04 |  |
| BRE | WWP1 | 17 | 141303 | 2,52E-03 |  |
| BRE | YBX1 | 41 | 141303 | 5,42E-07 | (*) |
| BRE | ZNF259 | 15 | 141303 | 5,10E-03 |  |
| CNS | ASF1B | 19 | 195516 | 0,00E+00 | (*) |
| CNS | AURKB | 13 | 195516 | 0,00E+00 | (*) |
| CNS | C17ORF79 | 10 | 195516 | 1,45E-07 | (*) |
| CNS | C1ORF2 | 13 | 195516 | 0,00E+00 | (*) |
| CNS | CDC45L | 11 | 195516 | 2,86E-08 | (*) |
| CNS | CDCA5 | 16 | 195516 | 0,00E+00 | (*) |
| CNS | CKS2 | 12 | 195516 | 6,33E-09 | (*) |
| CNS | COL20A1 | 26 | 195516 | 0,00E+00 | (*) |
| CNS | CSNK1G2 | 10 | 195516 | 1,45E-07 | (*) |
| CNS | DBH | 42 | 195516 | 0,00E+00 | (*) |
| CNS | DLK1 | 18 | 195516 | 0,00E+00 | (*) |
| CNS | ESPL1 | 10 | 195516 | 1,45E-07 | (*) |
| CNS | GATA2 | 19 | 195516 | 0,00E+00 | (*) |
| CNS | IRAK1 | 15 | 195516 | 0,00E+00 | (*) |
| CNS | KREMEN2 | 11 | 195516 | 2,86E-08 | (*) |
| CNS | KRT13 | 28 | 195516 | 0,00E+00 | (*) |
| CNS | KRT16 | 10 | 195516 | 1,45E-07 | (*) |
| CNS | KRT17 | 120 | 195516 | 0,00E+00 | (*) |
| CNS | KRT6A | 190 | 195516 | 0,00E+00 | (*) |
| CNS | NAPRT1 | 11 | 195516 | 2,86E-08 | (*) |
| CNS | PADI3 | 10 | 195516 | 1,45E-07 | (*) |
| CNS | PHOX2A | 11 | 195516 | 2,86E-08 | (*) |
| CNS | POLD1 | 16 | 195516 | 0,00E+00 | (*) |
| CNS | PRAME | 47 | 195516 | 9,63E-12 | (*) |
| CNS | PTBP1 | 33 | 195516 | 0,00E+00 | (*) |
| CNS | SHD | 16 | 195516 | 0,00E+00 | (*) |
| CNS | SLC6A2 | 13 | 195516 | 0,00E+00 | (*) |
| CNS | SPC25 | 11 | 195516 | 2,86E-08 | (*) |
| CNS | TFAP2B | 10 | 195516 | 1,45E-07 | (*) |
| CNS | TRABD | 14 | 195516 | 0,00E+00 | (*) |
| CNS | TSLP | 13 | 195516 | 0,00E+00 | (*) |
| COL | APEX1 | 33 | 184232 | 1,54E-03 |  |
| COL | APOBEC1 | 34 | 184232 | 1,27E-03 |  |
| COL | ARPC4 | 26 | 184232 | 6,10E-03 |  |
| COL | BAT2D1 | 28 | 184232 | 4,12E-03 |  |
| COL | BMP4 | 24 | 184232 | 9,03E-03 |  |
| COL | BMPR2 | 25 | 184232 | 7,42E-03 |  |
| COL | BRD2 | 26 | 184232 | 6,10E-03 |  |
| COL | C20ORF149 | 42 | 184232 | 2,64E-04 |  |
| COL | CD82 | 26 | 184232 | 6,10E-03 |  |
| COL | CDK4 | 27 | 184232 | 5,01E-03 |  |
| COL | CREB3L1 | 30 | 184232 | 2,78E-03 |  |
| COL | CSH2 | 33 | 184232 | 1,54E-03 |  |
| COL | DDB1 | 36 | 184232 | 8,58E-04 |  |
| COL | DNMT3A | 47 | 184232 | 9,91E-05 |  |
| COL | EIF3B | 42 | 184232 | 2,64E-04 |  |
| COL | HMGN2 | 41 | 184232 | 3,22E-04 |  |
| COL | KIAA0100 | 26 | 184232 | 6,10E-03 |  |
| COL | KLHDC2 | 31 | 184232 | 2,29E-03 |  |
| COL | LAPTM4A | 44 | 184232 | 1,79E-04 |  |
| COL | LGALS3BP | 85 | 184232 | 5,78E-08 | (*) |
| COL | MT1A | 40 | 184232 | 3,91E-04 |  |
| COL | MYO1B | 24 | 184232 | 9,03E-03 |  |
| COL | NQO1 | 33 | 184232 | 1,54E-03 |  |
| COL | PI3 | 27 | 184232 | 5,01E-03 |  |
| COL | PKP2 | 25 | 184232 | 7,42E-03 |  |
| COL | PPP1CA | 26 | 184232 | 6,10E-03 |  |
| COL | PSEN1 | 72 | 184232 | 7,35E-07 | (*) |
| COL | PSMA3 | 24 | 184232 | 9,03E-03 |  |
| COL | PTK2 | 25 | 184232 | 7,42E-03 |  |
| COL | RALY | 32 | 184232 | 1,88E-03 |  |
| COL | RPS7 | 33 | 184232 | 1,54E-03 |  |
| COL | SAE1 | 26 | 184232 | 6,10E-03 |  |
| COL | SEC24C | 28 | 184232 | 4,12E-03 |  |
| COL | SENP5 | 27 | 184232 | 5,01E-03 |  |
| COL | TK1 | 28 | 184232 | 4,12E-03 |  |
| COL | TNFRSF10B | 46 | 184232 | 1,21E-04 |  |
| COL | TPX2 | 29 | 184232 | 3,39E-03 |  |
| COL | YWHAZ | 24 | 184232 | 9,03E-03 |  |
| DER | ABT1 | 18 | 138449 | 6,89E-04 |  |
| DER | AEN | 24 | 138449 | 6,08E-05 |  |
| DER | AKT1S1 | 28 | 138449 | 1,21E-05 | (*) |
| DER | ALKBH7 | 14 | 138449 | 3,47E-03 |  |
| DER | APOD | 37 | 138449 | 3,16E-07 | (*) |
| DER | ARHGEF1 | 36 | 138449 | 4,74E-07 | (*) |
| DER | ARL6IP4 | 15 | 138449 | 2,32E-03 |  |
| DER | ARMET | 45 | 138449 | 1,27E-08 | (*) |
| DER | ASB9 | 12 | 138449 | 7,80E-03 |  |
| DER | ASF1B | 12 | 138449 | 7,80E-03 |  |
| DER | BAMBI | 14 | 138449 | 3,47E-03 |  |
| DER | BAP1 | 22 | 138449 | 1,37E-04 |  |
| DER | BAT2L | 14 | 138449 | 3,47E-03 |  |
| DER | BHLHE40 | 12 | 138449 | 7,80E-03 |  |
| DER | BIRC7 | 36 | 138449 | 4,74E-07 | (*) |
| DER | BRCA1 | 16 | 138449 | 1,55E-03 |  |
| DER | BRF1 | 21 | 138449 | 2,05E-04 |  |
| DER | BST2 | 21 | 138449 | 2,05E-04 |  |
| DER | BTBD2 | 17 | 138449 | 1,03E-03 |  |
| DER | C12ORF44 | 14 | 138449 | 3,47E-03 |  |
| DER | C13ORF18 | 13 | 138449 | 5,20E-03 |  |
| DER | C14ORF166 | 14 | 138449 | 3,47E-03 |  |
| DER | C16ORF68 | 15 | 138449 | 2,32E-03 |  |
| DER | C17ORF60 | 24 | 138449 | 6,08E-05 |  |
| DER | C17ORF63 | 20 | 138449 | 3,07E-04 |  |
| DER | C20ORF27 | 21 | 138449 | 2,05E-04 |  |
| DER | C2ORF7 | 12 | 138449 | 7,80E-03 |  |
| DER | C7ORF27 | 18 | 138449 | 6,89E-04 |  |
| DER | CAPS | 16 | 138449 | 1,55E-03 |  |
| DER | CCM2 | 16 | 138449 | 1,55E-03 |  |
| DER | CCNB1 | 60 | 138449 | 2,49E-10 | (*) |
| DER | CD320 | 22 | 138449 | 1,37E-04 |  |
| DER | CD68 | 29 | 138449 | 8,05E-06 | (*) |
| DER | CDC20 | 22 | 138449 | 1,37E-04 |  |
| DER | CDC45L | 13 | 138449 | 5,20E-03 |  |
| DER | CENPM | 20 | 138449 | 3,07E-04 |  |
| DER | CHMP6 | 14 | 138449 | 3,47E-03 |  |
| DER | COL9A3 | 29 | 138449 | 8,05E-06 | (*) |
| DER | COQ9 | 13 | 138449 | 5,20E-03 |  |
| DER | COTL1 | 21 | 138449 | 2,05E-04 |  |
| DER | COX4NB | 15 | 138449 | 2,32E-03 |  |
| DER | COX5B | 12 | 138449 | 7,80E-03 |  |
| DER | CPNE2 | 12 | 138449 | 7,80E-03 |  |
| DER | CXORF56 | 13 | 138449 | 5,20E-03 |  |
| DER | DAK | 18 | 138449 | 6,89E-04 |  |
| DER | DDX39 | 26 | 138449 | 2,71E-05 | (*) |
| DER | DENND2D | 12 | 138449 | 7,80E-03 |  |
| DER | DENND5A | 12 | 138449 | 7,80E-03 |  |
| DER | DHRS2 | 21 | 138449 | 2,05E-04 |  |
| DER | DHX37 | 13 | 138449 | 5,20E-03 |  |
| DER | DLL3 | 33 | 138449 | 1,60E-06 | (*) |
| DER | DNAJC8 | 25 | 138449 | 4,06E-05 |  |
| DER | DOHH | 13 | 138449 | 5,20E-03 |  |
| DER | DSTYK | 15 | 138449 | 2,32E-03 |  |
| DER | DTYMK | 13 | 138449 | 5,20E-03 |  |
| DER | DUSP23 | 15 | 138449 | 2,32E-03 |  |
| DER | EDEM2 | 26 | 138449 | 2,71E-05 | (*) |
| DER | EHBP1L1 | 14 | 138449 | 3,47E-03 |  |
| DER | EIF4EBP1 | 18 | 138449 | 6,89E-04 |  |
| DER | EMD | 46 | 138449 | 8,26E-09 | (*) |
| DER | ESRRA | 18 | 138449 | 6,89E-04 |  |
| DER | FADD | 14 | 138449 | 3,47E-03 |  |
| DER | FAM134A | 14 | 138449 | 3,47E-03 |  |
| DER | FASTK | 17 | 138449 | 1,03E-03 |  |
| DER | FHOD1 | 12 | 138449 | 7,80E-03 |  |
| DER | FTSJ3 | 16 | 138449 | 1,55E-03 |  |
| DER | FXYD3 | 18 | 138449 | 6,89E-04 |  |
| DER | G3BP2 | 14 | 138449 | 3,47E-03 |  |
| DER | G6PC3 | 26 | 138449 | 2,71E-05 | (*) |
| DER | GBL | 12 | 138449 | 7,80E-03 |  |
| DER | GIPC1 | 24 | 138449 | 6,08E-05 |  |
| DER | GLB1L3 | 15 | 138449 | 2,32E-03 |  |
| DER | GMPPB | 13 | 138449 | 5,20E-03 |  |
| DER | GPN2 | 21 | 138449 | 2,05E-04 |  |
| DER | GPR143 | 13 | 138449 | 5,20E-03 |  |
| DER | GPS2 | 30 | 138449 | 5,37E-06 | (*) |
| DER | GSDMD | 12 | 138449 | 7,80E-03 |  |
| DER | GYG2 | 12 | 138449 | 7,80E-03 |  |
| DER | GYPC | 20 | 138449 | 3,07E-04 |  |
| DER | HAUS8 | 17 | 138449 | 1,03E-03 |  |
| DER | HDAC3 | 14 | 138449 | 3,47E-03 |  |
| DER | HIRIP3 | 12 | 138449 | 7,80E-03 |  |
| DER | HMGXB3 | 20 | 138449 | 3,07E-04 |  |
| DER | HMHA1 | 18 | 138449 | 6,89E-04 |  |
| DER | IDI1 | 15 | 138449 | 2,32E-03 |  |
| DER | IFI30 | 18 | 138449 | 6,89E-04 |  |
| DER | IFI35 | 24 | 138449 | 6,08E-05 |  |
| DER | INTS1 | 26 | 138449 | 2,71E-05 | (*) |
| DER | IRAK1 | 49 | 138449 | 2,42E-09 | (*) |
| DER | ITIH5 | 20 | 138449 | 3,07E-04 |  |
| DER | ITPK1 | 16 | 138449 | 1,55E-03 |  |
| DER | KATNB1 | 27 | 138449 | 1,81E-05 | (*) |
| DER | KIAA1967 | 14 | 138449 | 3,47E-03 |  |
| DER | KIF7 | 14 | 138449 | 3,47E-03 |  |
| DER | KRT34 | 29 | 138449 | 8,05E-06 | (*) |
| DER | LEF1 | 20 | 138449 | 3,07E-04 |  |
| DER | LMNB2 | 12 | 138449 | 7,80E-03 |  |
| DER | LRWD1 | 15 | 138449 | 2,32E-03 |  |
| DER | MAGEA10 | 17 | 138449 | 1,03E-03 |  |
| DER | MAGEA6 | 31 | 138449 | 3,58E-06 | (*) |
| DER | MAGEC2 | 36 | 138449 | 4,74E-07 | (*) |
| DER | MARCKSL1 | 13 | 138449 | 5,20E-03 |  |
| DER | MAZ | 32 | 138449 | 2,39E-06 | (*) |
| DER | MBD3 | 19 | 138449 | 4,60E-04 |  |
| DER | MCM3AP | 25 | 138449 | 4,06E-05 |  |
| DER | MEN1 | 14 | 138449 | 3,47E-03 |  |
| DER | MFI2 | 20 | 138449 | 3,07E-04 |  |
| DER | MIA | 12 | 138449 | 7,80E-03 |  |
| DER | MPG | 20 | 138449 | 3,07E-04 |  |
| DER | MRPL24 | 22 | 138449 | 1,37E-04 |  |
| DER | MRPS12 | 17 | 138449 | 1,03E-03 |  |
| DER | MRTO4 | 25 | 138449 | 4,06E-05 |  |
| DER | MYO1D | 12 | 138449 | 7,80E-03 |  |
| DER | MYO9B | 20 | 138449 | 3,07E-04 |  |
| DER | NCAPH2 | 32 | 138449 | 2,39E-06 | (*) |
| DER | NCK2 | 52 | 138449 | 1,30E-09 | (*) |
| DER | NDUFA13 | 21 | 138449 | 2,05E-04 |  |
| DER | NEDD4L | 21 | 138449 | 2,05E-04 |  |
| DER | NOC4L | 20 | 138449 | 3,07E-04 |  |
| DER | NT5DC3 | 14 | 138449 | 3,47E-03 |  |
| DER | NUDT21 | 18 | 138449 | 6,89E-04 |  |
| DER | OGFR | 15 | 138449 | 2,32E-03 |  |
| DER | ORAI2 | 14 | 138449 | 3,47E-03 |  |
| DER | PACS2 | 17 | 138449 | 1,03E-03 |  |
| DER | PAGE5 | 15 | 138449 | 2,32E-03 |  |
| DER | PAK4 | 17 | 138449 | 1,03E-03 |  |
| DER | PARK7 | 21 | 138449 | 2,05E-04 |  |
| DER | PCDHGA1 | 15 | 138449 | 2,32E-03 |  |
| DER | PCDHGA10 | 15 | 138449 | 2,32E-03 |  |
| DER | PCDHGA11 | 15 | 138449 | 2,32E-03 |  |
| DER | PCDHGA12 | 15 | 138449 | 2,32E-03 |  |
| DER | PCDHGA2 | 15 | 138449 | 2,32E-03 |  |
| DER | PCDHGA3 | 15 | 138449 | 2,32E-03 |  |
| DER | PCDHGA4 | 15 | 138449 | 2,32E-03 |  |
| DER | PCDHGA5 | 15 | 138449 | 2,32E-03 |  |
| DER | PCDHGA6 | 15 | 138449 | 2,32E-03 |  |
| DER | PCDHGA7 | 15 | 138449 | 2,32E-03 |  |
| DER | PCDHGA8 | 15 | 138449 | 2,32E-03 |  |
| DER | PCDHGA9 | 15 | 138449 | 2,32E-03 |  |
| DER | PCDHGB1 | 15 | 138449 | 2,32E-03 |  |
| DER | PCDHGB2 | 15 | 138449 | 2,32E-03 |  |
| DER | PCDHGB3 | 15 | 138449 | 2,32E-03 |  |
| DER | PCDHGB4 | 15 | 138449 | 2,32E-03 |  |
| DER | PCDHGB5 | 15 | 138449 | 2,32E-03 |  |
| DER | PCDHGB6 | 15 | 138449 | 2,32E-03 |  |
| DER | PCDHGB7 | 15 | 138449 | 2,32E-03 |  |
| DER | PCDHGC3 | 15 | 138449 | 2,32E-03 |  |
| DER | PCDHGC4 | 15 | 138449 | 2,32E-03 |  |
| DER | PCDHGC5 | 15 | 138449 | 2,32E-03 |  |
| DER | PDE6D | 13 | 138449 | 5,20E-03 |  |
| DER | PDRG1 | 16 | 138449 | 1,55E-03 |  |
| DER | PFDN5 | 16 | 138449 | 1,55E-03 |  |
| DER | PFKP | 31 | 138449 | 3,58E-06 | (*) |
| DER | PHC2 | 27 | 138449 | 1,81E-05 | (*) |
| DER | PLCB3 | 13 | 138449 | 5,20E-03 |  |
| DER | PLEKHB1 | 28 | 138449 | 1,21E-05 | (*) |
| DER | PLP1 | 31 | 138449 | 3,58E-06 | (*) |
| DER | POLD1 | 12 | 138449 | 7,80E-03 |  |
| DER | POLR1E | 13 | 138449 | 5,20E-03 |  |
| DER | POLR3H | 13 | 138449 | 5,20E-03 |  |
| DER | POM121 | 16 | 138449 | 1,55E-03 |  |
| DER | PRAME | 101 | 138449 | 7,42E-11 | (*) |
| DER | PREX1 | 25 | 138449 | 4,06E-05 |  |
| DER | PRPF31 | 30 | 138449 | 5,37E-06 | (*) |
| DER | PRR14 | 31 | 138449 | 3,58E-06 | (*) |
| DER | PSCA | 16 | 138449 | 1,55E-03 |  |
| DER | PSMC1 | 28 | 138449 | 1,21E-05 | (*) |
| DER | PSMC3IP | 43 | 138449 | 2,83E-08 | (*) |
| DER | PSMD11 | 20 | 138449 | 3,07E-04 |  |
| DER | PSRC1 | 26 | 138449 | 2,71E-05 | (*) |
| DER | PTPN23 | 15 | 138449 | 2,32E-03 |  |
| DER | PYCR1 | 17 | 138449 | 1,03E-03 |  |
| DER | PYGB | 47 | 138449 | 5,77E-09 | (*) |
| DER | RABEP2 | 12 | 138449 | 7,80E-03 |  |
| DER | RBCK1 | 14 | 138449 | 3,47E-03 |  |
| DER | RDH13 | 14 | 138449 | 3,47E-03 |  |
| DER | RHBDD2 | 19 | 138449 | 4,60E-04 |  |
| DER | RLBP1 | 14 | 138449 | 3,47E-03 |  |
| DER | RNASEH2A | 34 | 138449 | 1,06E-06 | (*) |
| DER | RPL29 | 27 | 138449 | 1,81E-05 | (*) |
| DER | RPS19BP1 | 17 | 138449 | 1,03E-03 |  |
| DER | RPUSD1 | 15 | 138449 | 2,32E-03 |  |
| DER | S100A1 | 28 | 138449 | 1,21E-05 | (*) |
| DER | S100B | 58 | 138449 | 2,65E-10 | (*) |
| DER | SAAL1 | 24 | 138449 | 6,08E-05 |  |
| DER | SAMM50 | 39 | 138449 | 1,41E-07 | (*) |
| DER | SCCPDH | 14 | 138449 | 3,47E-03 |  |
| DER | SEC22B | 13 | 138449 | 5,20E-03 |  |
| DER | SEMA6A | 17 | 138449 | 1,03E-03 |  |
| DER | SERPINA3 | 30 | 138449 | 5,37E-06 | (*) |
| DER | SETDB1 | 13 | 138449 | 5,20E-03 |  |
| DER | SF3A2 | 23 | 138449 | 9,11E-05 |  |
| DER | SH3BP5 | 21 | 138449 | 2,05E-04 |  |
| DER | SIGMAR1 | 64 | 138449 | 2,01E-10 | (*) |
| DER | SIVA1 | 12 | 138449 | 7,80E-03 |  |
| DER | SLC39A11 | 19 | 138449 | 4,60E-04 |  |
| DER | SLC39A4 | 15 | 138449 | 2,32E-03 |  |
| DER | SLC44A4 | 12 | 138449 | 7,80E-03 |  |
| DER | SLC7A5 | 31 | 138449 | 3,58E-06 | (*) |
| DER | SMARCD1 | 13 | 138449 | 5,20E-03 |  |
| DER | SNRPA | 17 | 138449 | 1,03E-03 |  |
| DER | SOX10 | 42 | 138449 | 4,20E-08 | (*) |
| DER | SPHK1 | 15 | 138449 | 2,32E-03 |  |
| DER | SPP1 | 12 | 138449 | 7,80E-03 |  |
| DER | SRA1 | 13 | 138449 | 5,20E-03 |  |
| DER | STK10 | 14 | 138449 | 3,47E-03 |  |
| DER | STOML1 | 15 | 138449 | 2,32E-03 |  |
| DER | STRA13 | 19 | 138449 | 4,60E-04 |  |
| DER | SUPT4H1 | 13 | 138449 | 5,20E-03 |  |
| DER | SURF6 | 15 | 138449 | 2,32E-03 |  |
| DER | TBC1D16 | 15 | 138449 | 2,32E-03 |  |
| DER | TCOF1 | 30 | 138449 | 5,37E-06 | (*) |
| DER | TFPT | 14 | 138449 | 3,47E-03 |  |
| DER | TIMELESS | 20 | 138449 | 3,07E-04 |  |
| DER | TIMM13 | 20 | 138449 | 3,07E-04 |  |
| DER | TIMP2 | 21 | 138449 | 2,05E-04 |  |
| DER | TK1 | 34 | 138449 | 1,06E-06 | (*) |
| DER | TMC6 | 21 | 138449 | 2,05E-04 |  |
| DER | TMED1 | 19 | 138449 | 4,60E-04 |  |
| DER | TMEM141 | 16 | 138449 | 1,55E-03 |  |
| DER | TMEM167A | 14 | 138449 | 3,47E-03 |  |
| DER | TNC | 17 | 138449 | 1,03E-03 |  |
| DER | TNFRSF14 | 41 | 138449 | 6,26E-08 | (*) |
| DER | TRIB3 | 17 | 138449 | 1,03E-03 |  |
| DER | TROAP | 17 | 138449 | 1,03E-03 |  |
| DER | TSPAN10 | 18 | 138449 | 6,89E-04 |  |
| DER | TSSC4 | 19 | 138449 | 4,60E-04 |  |
| DER | TUBB2A | 13 | 138449 | 5,20E-03 |  |
| DER | TUBB4 | 20 | 138449 | 3,07E-04 |  |
| DER | TYMS | 12 | 138449 | 7,80E-03 |  |
| DER | UBE2C | 12 | 138449 | 7,80E-03 |  |
| DER | UBE2S | 28 | 138449 | 1,21E-05 | (*) |
| DER | UPP1 | 52 | 138449 | 1,30E-09 | (*) |
| DER | UQCRFS1 | 12 | 138449 | 7,80E-03 |  |
| DER | UQCRQ | 16 | 138449 | 1,55E-03 |  |
| DER | USP5 | 32 | 138449 | 2,39E-06 | (*) |
| DER | UTP18 | 17 | 138449 | 1,03E-03 |  |
| DER | WDR74 | 14 | 138449 | 3,47E-03 |  |
| DER | XRCC1 | 14 | 138449 | 3,47E-03 |  |
| DER | XYLT2 | 13 | 138449 | 5,20E-03 |  |
| DER | YIPF2 | 29 | 138449 | 8,05E-06 | (*) |
| DER | ZC3HC1 | 12 | 138449 | 7,80E-03 |  |
| DER | ZDHHC16 | 12 | 138449 | 7,80E-03 |  |
| DER | ZNF259 | 14 | 138449 | 3,47E-03 |  |
| DER | ZNRD1 | 13 | 138449 | 5,20E-03 |  |
| END | CCL8 | 10 | 113927 | 1,39E-04 |  |
| END | CCNB2 | 10 | 113927 | 1,39E-04 |  |
| END | CSH2 | 12 | 113927 | 2,35E-05 | (*) |
| END | CYFIP1 | 10 | 113927 | 1,39E-04 |  |
| END | EML4 | 10 | 113927 | 1,39E-04 |  |
| END | G3BP2 | 12 | 113927 | 2,35E-05 | (*) |
| END | HBG2 | 22 | 113927 | 2,81E-09 | (*) |
| END | MAPKAPK3 | 25 | 113927 | 3,58E-12 | (*) |
| END | MSMP | 10 | 113927 | 1,39E-04 |  |
| END | MSN | 11 | 113927 | 5,72E-05 |  |
| END | MYO19 | 12 | 113927 | 2,35E-05 | (*) |
| END | NPFF | 10 | 113927 | 1,39E-04 |  |
| END | PCSK5 | 13 | 113927 | 9,69E-06 | (*) |
| END | RNASE1 | 12 | 113927 | 2,35E-05 | (*) |
| END | RPA1 | 10 | 113927 | 1,39E-04 |  |
| END | TUBD1 | 19 | 113927 | 4,67E-08 | (*) |
| EYE | ATF5 | 20 | 51699 | 0,00E+00 | (*) |
| EYE | BLCAP | 10 | 51699 | 1,12E-06 | (*) |
| EYE | CHGA | 11 | 51699 | 2,84E-07 | (*) |
| EYE | CNP | 10 | 51699 | 1,12E-06 | (*) |
| EYE | KIAA0101 | 12 | 51699 | 7,26E-08 | (*) |
| EYE | MYCN | 11 | 51699 | 2,84E-07 | (*) |
| EYE | POLE3 | 23 | 51699 | 0,00E+00 | (*) |
| EYE | TMEM97 | 10 | 51699 | 1,12E-06 | (*) |
| EYE | TOMM40 | 14 | 51699 | 4,79E-09 | (*) |
| EYE | WDTC1 | 10 | 51699 | 1,12E-06 | (*) |
| INT | ADH7 | 28 | 33535 | 9,91E-09 | (*) |
| INT | ANLN | 11 | 33535 | 7,18E-04 |  |
| INT | ATF6 | 16 | 33535 | 2,67E-05 | (*) |
| INT | ATP6V0C | 10 | 33535 | 1,39E-03 |  |
| INT | CCNB2 | 13 | 33535 | 1,93E-04 |  |
| INT | CDC123 | 11 | 33535 | 7,18E-04 |  |
| INT | CNN3 | 10 | 33535 | 1,39E-03 |  |
| INT | DKK1 | 20 | 33535 | 1,92E-06 | (*) |
| INT | EEF2 | 26 | 33535 | 3,70E-08 | (*) |
| INT | EIF5A | 11 | 33535 | 7,18E-04 |  |
| INT | GCG | 103 | 33535 | 7,27E-11 | (*) |
| INT | GNAS | 11 | 33535 | 7,18E-04 |  |
| INT | IFIT1 | 10 | 33535 | 1,39E-03 |  |
| INT | ILF2 | 11 | 33535 | 7,18E-04 |  |
| INT | KRT13 | 23 | 33535 | 2,67E-07 | (*) |
| INT | KRT17 | 44 | 33535 | 0,00E+00 | (*) |
| INT | KRT18 | 74 | 33535 | 9,07E-11 | (*) |
| INT | KRT19 | 10 | 33535 | 1,39E-03 |  |
| INT | LAPTM4B | 13 | 33535 | 1,93E-04 |  |
| INT | LGALS3BP | 20 | 33535 | 1,92E-06 | (*) |
| INT | LMAN1 | 11 | 33535 | 7,18E-04 |  |
| INT | MAGEA4 | 17 | 33535 | 1,38E-05 | (*) |
| INT | MMADHC | 51 | 33535 | 1,13E-10 | (*) |
| INT | MMP10 | 31 | 33535 | 1,36E-09 | (*) |
| INT | OLR1 | 64 | 33535 | 6,64E-11 | (*) |
| INT | PPIB | 10 | 33535 | 1,39E-03 |  |
| INT | PRKAR1A | 11 | 33535 | 7,18E-04 |  |
| INT | PRNP | 14 | 33535 | 9,98E-05 |  |
| INT | PRPF6 | 11 | 33535 | 7,18E-04 |  |
| INT | PSMB5 | 12 | 33535 | 3,72E-04 |  |
| INT | PSMC3 | 15 | 33535 | 5,17E-05 |  |
| INT | RPL7 | 13 | 33535 | 1,93E-04 |  |
| INT | SERPINB2 | 14 | 33535 | 9,98E-05 |  |
| INT | SLC2A1 | 28 | 33535 | 9,91E-09 | (*) |
| INT | SSR2 | 17 | 33535 | 1,38E-05 | (*) |
| INT | STOML2 | 10 | 33535 | 1,39E-03 |  |
| INT | THBS1 | 45 | 33535 | 0,00E+00 | (*) |
| INT | TMPRSS11B | 10 | 33535 | 1,39E-03 |  |
| INT | TRIM16 | 15 | 33535 | 5,17E-05 |  |
| INT | TRIM16L | 28 | 33535 | 9,91E-09 | (*) |
| INT | VDAC2 | 10 | 33535 | 1,39E-03 |  |
| KID | ABCA2 | 16 | 85801 | 2,57E-06 | (*) |
| KID | ACTC1 | 22 | 85801 | 2,09E-08 | (*) |
| KID | AIFM3 | 10 | 85801 | 3,21E-04 |  |
| KID | ARPC1B | 18 | 85801 | 5,14E-07 | (*) |
| KID | BCAM | 10 | 85801 | 3,21E-04 |  |
| KID | BCAS1 | 23 | 85801 | 9,63E-09 | (*) |
| KID | BZW2 | 10 | 85801 | 3,21E-04 |  |
| KID | C19ORF10 | 10 | 85801 | 3,21E-04 |  |
| KID | C20ORF149 | 15 | 85801 | 5,74E-06 | (*) |
| KID | C3 | 16 | 85801 | 2,57E-06 | (*) |
| KID | CAMKV | 19 | 85801 | 2,30E-07 | (*) |
| KID | CCDC72 | 10 | 85801 | 3,21E-04 |  |
| KID | CCNB1 | 19 | 85801 | 2,30E-07 | (*) |
| KID | CD163 | 19 | 85801 | 2,30E-07 | (*) |
| KID | CHGB | 11 | 85801 | 1,43E-04 |  |
| KID | COX6B1 | 15 | 85801 | 5,74E-06 | (*) |
| KID | CRYAB | 19 | 85801 | 2,30E-07 | (*) |
| KID | EIF4H | 11 | 85801 | 1,43E-04 |  |
| KID | EIF6 | 10 | 85801 | 3,21E-04 |  |
| KID | ELMO1 | 17 | 85801 | 1,15E-06 | (*) |
| KID | ERMN | 13 | 85801 | 2,87E-05 | (*) |
| KID | FLOT1 | 15 | 85801 | 5,74E-06 | (*) |
| KID | G6PD | 15 | 85801 | 5,74E-06 | (*) |
| KID | GUK1 | 12 | 85801 | 6,42E-05 |  |
| KID | HMP19 | 25 | 85801 | 2,18E-09 | (*) |
| KID | IRAK1 | 17 | 85801 | 1,15E-06 | (*) |
| KID | KRT19 | 16 | 85801 | 2,57E-06 | (*) |
| KID | MAG | 73 | 85801 | 3,46E-10 | (*) |
| KID | MGP | 13 | 85801 | 2,87E-05 | (*) |
| KID | MOBP | 22 | 85801 | 2,09E-08 | (*) |
| KID | MOG | 14 | 85801 | 1,28E-05 | (*) |
| KID | P2RY12 | 10 | 85801 | 3,21E-04 |  |
| KID | PACS1 | 11 | 85801 | 1,43E-04 |  |
| KID | PAQR6 | 31 | 85801 | 6,14E-10 | (*) |
| KID | PLEKHM2 | 11 | 85801 | 1,43E-04 |  |
| KID | PMP2 | 14 | 85801 | 1,28E-05 | (*) |
| KID | PTPRF | 12 | 85801 | 6,42E-05 |  |
| KID | RBCK1 | 11 | 85801 | 1,43E-04 |  |
| KID | RHOC | 15 | 85801 | 5,74E-06 | (*) |
| KID | RPS2 | 31 | 85801 | 6,14E-10 | (*) |
| KID | RPS5 | 40 | 85801 | 4,53E-10 | (*) |
| KID | SCG2 | 29 | 85801 | 7,47E-10 | (*) |
| KID | SERPINA3 | 15 | 85801 | 5,74E-06 | (*) |
| KID | SLC1A5 | 17 | 85801 | 1,15E-06 | (*) |
| KID | SLC38A5 | 15 | 85801 | 5,74E-06 | (*) |
| KID | SRGAP1 | 10 | 85801 | 3,21E-04 |  |
| KID | SYNPR | 24 | 85801 | 4,38E-09 | (*) |
| KID | TF | 135 | 85801 | 4,48E-10 | (*) |
| KID | TMEM144 | 10 | 85801 | 3,21E-04 |  |
| KID | TNFRSF12A | 12 | 85801 | 6,42E-05 |  |
| KID | TRIB3 | 10 | 85801 | 3,21E-04 |  |
| KID | TRIOBP | 11 | 85801 | 1,43E-04 |  |
| LIV | ADIPOR1 | 11 | 106121 | 1,02E-04 |  |
| LIV | AHSA1 | 17 | 106121 | 6,81E-07 | (*) |
| LIV | AKR1B10 | 12 | 106121 | 4,44E-05 |  |
| LIV | ANXA11 | 27 | 106121 | 0,00E+00 | (*) |
| LIV | APEH | 11 | 106121 | 1,02E-04 |  |
| LIV | ARL2 | 11 | 106121 | 1,02E-04 |  |
| LIV | ASPSCR1 | 21 | 106121 | 2,38E-08 | (*) |
| LIV | ATXN10 | 14 | 106121 | 8,35E-06 | (*) |
| LIV | BANP | 10 | 106121 | 2,36E-04 |  |
| LIV | BAT2 | 11 | 106121 | 1,02E-04 |  |
| LIV | BCL2L1 | 25 | 106121 | 6,01E-10 | (*) |
| LIV | BFSP1 | 10 | 106121 | 2,36E-04 |  |
| LIV | BPNT1 | 11 | 106121 | 1,02E-04 |  |
| LIV | BYSL | 11 | 106121 | 1,02E-04 |  |
| LIV | C10ORF2 | 12 | 106121 | 4,44E-05 |  |
| LIV | C10ORF54 | 57 | 106121 | 3,07E-10 | (*) |
| LIV | C13ORF1 | 11 | 106121 | 1,02E-04 |  |
| LIV | C19ORF62 | 23 | 106121 | 4,34E-09 | (*) |
| LIV | C1ORF77 | 39 | 106121 | 0,00E+00 | (*) |
| LIV | C20ORF149 | 10 | 106121 | 2,36E-04 |  |
| LIV | C6ORF192 | 20 | 106121 | 5,50E-08 | (*) |
| LIV | CAPN2 | 23 | 106121 | 4,34E-09 | (*) |
| LIV | CCNB1IP1 | 13 | 106121 | 1,92E-05 | (*) |
| LIV | CDC42 | 10 | 106121 | 2,36E-04 |  |
| LIV | CHTF18 | 13 | 106121 | 1,92E-05 | (*) |
| LIV | CLNS1A | 17 | 106121 | 6,81E-07 | (*) |
| LIV | COPE | 22 | 106121 | 1,02E-08 | (*) |
| LIV | CPSF3 | 14 | 106121 | 8,35E-06 | (*) |
| LIV | CRLF1 | 25 | 106121 | 6,01E-10 | (*) |
| LIV | DDX39 | 15 | 106121 | 3,62E-06 | (*) |
| LIV | DDX54 | 16 | 106121 | 1,57E-06 | (*) |
| LIV | DHRS2 | 98 | 106121 | 0,00E+00 | (*) |
| LIV | DHX15 | 13 | 106121 | 1,92E-05 | (*) |
| LIV | DKK1 | 15 | 106121 | 3,62E-06 | (*) |
| LIV | EPCAM | 15 | 106121 | 3,62E-06 | (*) |
| LIV | ERBB3 | 14 | 106121 | 8,35E-06 | (*) |
| LIV | FASN | 30 | 106121 | 0,00E+00 | (*) |
| LIV | FDXR | 10 | 106121 | 2,36E-04 |  |
| LIV | FGFR1 | 12 | 106121 | 4,44E-05 |  |
| LIV | FGFR4 | 11 | 106121 | 1,02E-04 |  |
| LIV | FKBP1A | 16 | 106121 | 1,57E-06 | (*) |
| LIV | FKBP8 | 27 | 106121 | 0,00E+00 | (*) |
| LIV | FLJ11506 | 10 | 106121 | 2,36E-04 |  |
| LIV | FLNA | 27 | 106121 | 0,00E+00 | (*) |
| LIV | FOSL1 | 34 | 106121 | 0,00E+00 | (*) |
| LIV | GDF15 | 35 | 106121 | 0,00E+00 | (*) |
| LIV | GMPS | 15 | 106121 | 3,62E-06 | (*) |
| LIV | GNB1 | 22 | 106121 | 1,02E-08 | (*) |
| LIV | GNB2 | 15 | 106121 | 3,62E-06 | (*) |
| LIV | GPRC5A | 11 | 106121 | 1,02E-04 |  |
| LIV | GSK3A | 18 | 106121 | 2,96E-07 | (*) |
| LIV | HYOU1 | 54 | 106121 | 0,00E+00 | (*) |
| LIV | IER3 | 13 | 106121 | 1,92E-05 | (*) |
| LIV | IFRD2 | 11 | 106121 | 1,02E-04 |  |
| LIV | IGBP1 | 10 | 106121 | 2,36E-04 |  |
| LIV | IGF2BP1 | 14 | 106121 | 8,35E-06 | (*) |
| LIV | IL1B | 20 | 106121 | 5,50E-08 | (*) |
| LIV | INF2 | 10 | 106121 | 2,36E-04 |  |
| LIV | IPO4 | 10 | 106121 | 2,36E-04 |  |
| LIV | ISCA1 | 14 | 106121 | 8,35E-06 | (*) |
| LIV | ITGA3 | 13 | 106121 | 1,92E-05 | (*) |
| LIV | IVNS1ABP | 15 | 106121 | 3,62E-06 | (*) |
| LIV | KRT10 | 39 | 106121 | 0,00E+00 | (*) |
| LIV | LAPTM4B | 13 | 106121 | 1,92E-05 | (*) |
| LIV | LARS2 | 15 | 106121 | 3,62E-06 | (*) |
| LIV | LTBP4 | 11 | 106121 | 1,02E-04 |  |
| LIV | MAL2 | 11 | 106121 | 1,02E-04 |  |
| LIV | MAN2A1 | 11 | 106121 | 1,02E-04 |  |
| LIV | MAP7D2 | 17 | 106121 | 6,81E-07 | (*) |
| LIV | METTL13 | 12 | 106121 | 4,44E-05 |  |
| LIV | MRPS26 | 17 | 106121 | 6,81E-07 | (*) |
| LIV | MYH9 | 32 | 106121 | 1,29E-10 | (*) |
| LIV | MYL9 | 15 | 106121 | 3,62E-06 | (*) |
| LIV | MYO18A | 11 | 106121 | 1,02E-04 |  |
| LIV | MYO1C | 15 | 106121 | 3,62E-06 | (*) |
| LIV | MYO1E | 10 | 106121 | 2,36E-04 |  |
| LIV | NALCN | 12 | 106121 | 4,44E-05 |  |
| LIV | NEDD4L | 16 | 106121 | 1,57E-06 | (*) |
| LIV | NINJ1 | 21 | 106121 | 2,38E-08 | (*) |
| LIV | NSUN5 | 11 | 106121 | 1,02E-04 |  |
| LIV | PFKM | 10 | 106121 | 2,36E-04 |  |
| LIV | PIH1D1 | 11 | 106121 | 1,02E-04 |  |
| LIV | PPP1R11 | 14 | 106121 | 8,35E-06 | (*) |
| LIV | PRAF2 | 12 | 106121 | 4,44E-05 |  |
| LIV | PREB | 13 | 106121 | 1,92E-05 | (*) |
| LIV | PRMT1 | 16 | 106121 | 1,57E-06 | (*) |
| LIV | PSMD4 | 12 | 106121 | 4,44E-05 |  |
| LIV | PTPN18 | 10 | 106121 | 2,36E-04 |  |
| LIV | RAB26 | 10 | 106121 | 2,36E-04 |  |
| LIV | RAGE | 21 | 106121 | 2,38E-08 | (*) |
| LIV | RBCK1 | 15 | 106121 | 3,62E-06 | (*) |
| LIV | RGNEF | 10 | 106121 | 2,36E-04 |  |
| LIV | RHOG | 10 | 106121 | 2,36E-04 |  |
| LIV | RIC8A | 18 | 106121 | 2,96E-07 | (*) |
| LIV | RNF8 | 12 | 106121 | 4,44E-05 |  |
| LIV | RPS2 | 13 | 106121 | 1,92E-05 | (*) |
| LIV | S100A11 | 11 | 106121 | 1,02E-04 |  |
| LIV | SDHC | 19 | 106121 | 1,28E-07 | (*) |
| LIV | SETD2 | 13 | 106121 | 1,92E-05 | (*) |
| LIV | SF3B4 | 10 | 106121 | 2,36E-04 |  |
| LIV | SHKBP1 | 11 | 106121 | 1,02E-04 |  |
| LIV | SLC25A1 | 15 | 106121 | 3,62E-06 | (*) |
| LIV | SLC2A3 | 18 | 106121 | 2,96E-07 | (*) |
| LIV | SLC38A1 | 11 | 106121 | 1,02E-04 |  |
| LIV | SREBF1 | 10 | 106121 | 2,36E-04 |  |
| LIV | SRP68 | 13 | 106121 | 1,92E-05 | (*) |
| LIV | STXBP2 | 11 | 106121 | 1,02E-04 |  |
| LIV | TADA3L | 17 | 106121 | 6,81E-07 | (*) |
| LIV | TAX1BP3 | 27 | 106121 | 0,00E+00 | (*) |
| LIV | TBC1D10B | 10 | 106121 | 2,36E-04 |  |
| LIV | TBC1D17 | 12 | 106121 | 4,44E-05 |  |
| LIV | TIRAP | 12 | 106121 | 4,44E-05 |  |
| LIV | TMEM8 | 14 | 106121 | 8,35E-06 | (*) |
| LIV | TMEM87A | 19 | 106121 | 1,28E-07 | (*) |
| LIV | TOMM34 | 11 | 106121 | 1,02E-04 |  |
| LIV | TRIOBP | 12 | 106121 | 4,44E-05 |  |
| LIV | TRMT1 | 13 | 106121 | 1,92E-05 | (*) |
| LIV | TRPC4AP | 10 | 106121 | 2,36E-04 |  |
| LIV | TRPV2 | 10 | 106121 | 2,36E-04 |  |
| LIV | TSPAN3 | 10 | 106121 | 2,36E-04 |  |
| LIV | TUBA1A | 17 | 106121 | 6,81E-07 | (*) |
| LIV | TXNRD2 | 11 | 106121 | 1,02E-04 |  |
| LIV | UCHL1 | 20 | 106121 | 5,50E-08 | (*) |
| LIV | USP14 | 27 | 106121 | 0,00E+00 | (*) |
| LIV | USP4 | 12 | 106121 | 4,44E-05 |  |
| LIV | USP7 | 14 | 106121 | 8,35E-06 | (*) |
| LIV | VASP | 11 | 106121 | 1,02E-04 |  |
| LIV | VIL1 | 21 | 106121 | 2,38E-08 | (*) |
| LIV | VPS25 | 14 | 106121 | 8,35E-06 | (*) |
| LIV | WDR70 | 12 | 106121 | 4,44E-05 |  |
| LIV | XPO5 | 15 | 106121 | 3,62E-06 | (*) |
| LIV | ZNF259 | 12 | 106121 | 4,44E-05 |  |
| LYM | ALG3 | 21 | 68796 | 3,12E-09 | (*) |
| LYM | ATAD3A | 16 | 68796 | 3,34E-07 | (*) |
| LYM | C21ORF33 | 17 | 68796 | 1,32E-07 | (*) |
| LYM | CDCA5 | 14 | 68796 | 2,15E-06 | (*) |
| LYM | CHMP4B | 12 | 68796 | 1,39E-05 | (*) |
| LYM | CPSF4 | 10 | 68796 | 8,96E-05 |  |
| LYM | ECSIT | 12 | 68796 | 1,39E-05 | (*) |
| LYM | FCGBP | 13 | 68796 | 5,47E-06 | (*) |
| LYM | FGFBP1 | 18 | 68796 | 5,18E-08 | (*) |
| LYM | FOXM1 | 25 | 68796 | 2,54E-10 | (*) |
| LYM | JTV1 | 10 | 68796 | 8,96E-05 |  |
| LYM | KIFC1 | 14 | 68796 | 2,15E-06 | (*) |
| LYM | KLK5 | 12 | 68796 | 1,39E-05 | (*) |
| LYM | KLK6 | 50 | 68796 | 0,00E+00 | (*) |
| LYM | KRT19 | 13 | 68796 | 5,47E-06 | (*) |
| LYM | KRT8 | 19 | 68796 | 2,03E-08 | (*) |
| LYM | LLGL1 | 40 | 68796 | 2,23E-11 | (*) |
| LYM | MDH1 | 17 | 68796 | 1,32E-07 | (*) |
| LYM | MRPL42 | 10 | 68796 | 8,96E-05 |  |
| LYM | NIPSNAP1 | 10 | 68796 | 8,96E-05 |  |
| LYM | NLRP7 | 11 | 68796 | 3,53E-05 | (*) |
| LYM | NUAK2 | 18 | 68796 | 5,18E-08 | (*) |
| LYM | NUDT1 | 15 | 68796 | 8,48E-07 | (*) |
| LYM | PPP1R16A | 12 | 68796 | 1,39E-05 | (*) |
| LYM | PRPF19 | 10 | 68796 | 8,96E-05 |  |
| LYM | PSAT1 | 33 | 68796 | 2,18E-10 | (*) |
| LYM | PYCR1 | 35 | 68796 | 0,00E+00 | (*) |
| LYM | RAB1B | 11 | 68796 | 3,53E-05 | (*) |
| LYM | RPS6KB2 | 14 | 68796 | 2,15E-06 | (*) |
| LYM | SCRIB | 10 | 68796 | 8,96E-05 |  |
| LYM | SLC28A1 | 12 | 68796 | 1,39E-05 | (*) |
| LYM | SLC38A10 | 27 | 68796 | 2,01E-10 | (*) |
| LYM | SLC7A5 | 15 | 68796 | 8,48E-07 | (*) |
| LYM | SNRPD3 | 10 | 68796 | 8,96E-05 |  |
| LYM | SSBP4 | 14 | 68796 | 2,15E-06 | (*) |
| LYM | STARD7 | 14 | 68796 | 2,15E-06 | (*) |
| LYM | SUPT16H | 11 | 68796 | 3,53E-05 | (*) |
| LYM | TMEM187 | 10 | 68796 | 8,96E-05 |  |
| LYM | ZNF689 | 41 | 68796 | 2,73E-11 | (*) |
| MSK | ADRBK1 | 10 | 94379 | 2,05E-04 |  |
| MSK | AIP | 11 | 94379 | 8,77E-05 |  |
| MSK | ARF5 | 12 | 94379 | 3,75E-05 |  |
| MSK | ATN1 | 10 | 94379 | 2,05E-04 |  |
| MSK | AURKB | 10 | 94379 | 2,05E-04 |  |
| MSK | C20ORF149 | 17 | 94379 | 5,37E-07 | (*) |
| MSK | C3ORF1 | 17 | 94379 | 5,37E-07 | (*) |
| MSK | CALR | 16 | 94379 | 1,26E-06 | (*) |
| MSK | CCNB1IP1 | 20 | 94379 | 4,23E-08 | (*) |
| MSK | CDH15 | 27 | 94379 | 2,67E-10 | (*) |
| MSK | CRELD2 | 12 | 94379 | 3,75E-05 |  |
| MSK | CXXC1 | 10 | 94379 | 2,05E-04 |  |
| MSK | DENND5A | 10 | 94379 | 2,05E-04 |  |
| MSK | DTYMK | 13 | 94379 | 1,60E-05 | (*) |
| MSK | EFHD1 | 14 | 94379 | 6,86E-06 | (*) |
| MSK | GDF15 | 21 | 94379 | 1,87E-08 | (*) |
| MSK | GIPC1 | 14 | 94379 | 6,86E-06 | (*) |
| MSK | GLI1 | 17 | 94379 | 5,37E-07 | (*) |
| MSK | HGS | 11 | 94379 | 8,77E-05 |  |
| MSK | INHBE | 17 | 94379 | 5,37E-07 | (*) |
| MSK | KATNB1 | 22 | 94379 | 8,17E-09 | (*) |
| MSK | KRT81 | 36 | 94379 | 7,57E-10 | (*) |
| MSK | MBD3 | 10 | 94379 | 2,05E-04 |  |
| MSK | MGAT4B | 17 | 94379 | 5,37E-07 | (*) |
| MSK | MPRIP | 17 | 94379 | 5,37E-07 | (*) |
| MSK | MYBL2 | 23 | 94379 | 3,84E-09 | (*) |
| MSK | MYOD1 | 24 | 94379 | 1,44E-09 | (*) |
| MSK | NRM | 10 | 94379 | 2,05E-04 |  |
| MSK | NTN5 | 18 | 94379 | 2,30E-07 | (*) |
| MSK | OTUD5 | 14 | 94379 | 6,86E-06 | (*) |
| MSK | PIPOX | 14 | 94379 | 6,86E-06 | (*) |
| MSK | PLEKHM2 | 11 | 94379 | 8,77E-05 |  |
| MSK | PREX1 | 23 | 94379 | 3,84E-09 | (*) |
| MSK | PSMB3 | 14 | 94379 | 6,86E-06 | (*) |
| MSK | RAB11B | 11 | 94379 | 8,77E-05 |  |
| MSK | SFTPC | 233 | 94379 | 7,28E-10 | (*) |
| MSK | SLC25A22 | 11 | 94379 | 8,77E-05 |  |
| MSK | SMPD4 | 10 | 94379 | 2,05E-04 |  |
| MSK | SMUG1 | 11 | 94379 | 8,77E-05 |  |
| MSK | SSRP1 | 19 | 94379 | 9,83E-08 | (*) |
| MSK | STK40 | 12 | 94379 | 3,75E-05 |  |
| MSK | TRABD | 11 | 94379 | 8,77E-05 |  |
| MSK | TRIB3 | 21 | 94379 | 1,87E-08 | (*) |
| MSK | UBE2S | 16 | 94379 | 1,26E-06 | (*) |
| MSK | WDR34 | 10 | 94379 | 2,05E-04 |  |
| MSK | WNT5B | 14 | 94379 | 6,86E-06 | (*) |
| PLA | ASNS | 13 | 43818 | 1,45E-09 | (*) |
| PLA | C12ORF41 | 13 | 43818 | 1,45E-09 | (*) |
| PLA | CAMTA2 | 11 | 43818 | 1,46E-08 | (*) |
| PLA | COL9A3 | 45 | 43818 | 1,12E-09 | (*) |
| PLA | DNMT3L | 34 | 43818 | 1,16E-09 | (*) |
| PLA | FPGS | 14 | 43818 | 8,35E-10 | (*) |
| PLA | GPKOW | 11 | 43818 | 1,46E-08 | (*) |
| PLA | HMGB2 | 19 | 43818 | 5,93E-10 | (*) |
| PLA | MAGEA6 | 20 | 43818 | 9,44E-10 | (*) |
| PLA | MRPL37 | 18 | 43818 | 6,32E-10 | (*) |
| PLA | SDCCAG3 | 10 | 43818 | 7,43E-08 | (*) |
| PLA | SF3A2 | 10 | 43818 | 7,43E-08 | (*) |
| PLA | SNX24 | 10 | 43818 | 7,43E-08 | (*) |
| PLA | TRMT2B | 10 | 43818 | 7,43E-08 | (*) |
| PLA | UBE2S | 10 | 43818 | 7,43E-08 | (*) |
| PLA | WDR18 | 11 | 43818 | 1,46E-08 | (*) |
| PRO | AGRN | 34 | 232720 | 2,68E-04 |  |
| PRO | C5ORF37 | 93 | 232720 | 0,00E+00 | (*) |
| PRO | CCNB1 | 34 | 232720 | 2,68E-04 |  |
| PRO | CCT6A | 28 | 232720 | 1,14E-03 |  |
| PRO | CTSD | 33 | 232720 | 3,41E-04 |  |
| PRO | EDC3 | 93 | 232720 | 0,00E+00 | (*) |
| PRO | EEF1A2 | 25 | 232720 | 2,36E-03 |  |
| PRO | ERCC1 | 29 | 232720 | 8,98E-04 |  |
| PRO | ERI2 | 20 | 232720 | 7,92E-03 |  |
| PRO | G6PD | 28 | 232720 | 1,14E-03 |  |
| PRO | ITGA3 | 39 | 232720 | 7,99E-05 |  |
| PRO | S100A16 | 26 | 232720 | 1,86E-03 |  |
| PRO | SPINT2 | 35 | 232720 | 2,10E-04 |  |
| PRO | SYNGR2 | 23 | 232720 | 3,83E-03 |  |
| RES | AARS2 | 11 | 145698 | 5,59E-03 |  |
| RES | ACY1 | 11 | 145698 | 5,59E-03 |  |
| RES | ADAMTS8 | 20 | 145698 | 8,01E-05 |  |
| RES | ADD1 | 10 | 145698 | 8,95E-03 |  |
| RES | AGPAT6 | 10 | 145698 | 8,95E-03 |  |
| RES | AIP | 13 | 145698 | 2,18E-03 |  |
| RES | AKR1B10 | 59 | 145698 | 0,00E+00 | (*) |
| RES | ALDH3B1 | 27 | 145698 | 2,95E-06 | (*) |
| RES | ANAPC2 | 11 | 145698 | 5,59E-03 |  |
| RES | AP2A1 | 18 | 145698 | 2,06E-04 |  |
| RES | APEH | 16 | 145698 | 5,29E-04 |  |
| RES | ARHGAP1 | 10 | 145698 | 8,95E-03 |  |
| RES | ARMC6 | 12 | 145698 | 3,49E-03 |  |
| RES | ARMET | 12 | 145698 | 3,49E-03 |  |
| RES | ARPC5L | 15 | 145698 | 8,47E-04 |  |
| RES | ATN1 | 10 | 145698 | 8,95E-03 |  |
| RES | ATP2C2 | 13 | 145698 | 2,18E-03 |  |
| RES | ATP5D | 15 | 145698 | 8,47E-04 |  |
| RES | ATP6V0C | 24 | 145698 | 1,21E-05 | (*) |
| RES | AURKB | 15 | 145698 | 8,47E-04 |  |
| RES | BAP1 | 10 | 145698 | 8,95E-03 |  |
| RES | BCL2L1 | 28 | 145698 | 1,84E-06 | (*) |
| RES | BOP1 | 17 | 145698 | 3,30E-04 |  |
| RES | BTBD2 | 15 | 145698 | 8,47E-04 |  |
| RES | BYSL | 13 | 145698 | 2,18E-03 |  |
| RES | C12ORF44 | 20 | 145698 | 8,01E-05 |  |
| RES | C16ORF45 | 14 | 145698 | 1,36E-03 |  |
| RES | C17ORF79 | 11 | 145698 | 5,59E-03 |  |
| RES | C1QBP | 25 | 145698 | 7,58E-06 | (*) |
| RES | C20ORF27 | 13 | 145698 | 2,18E-03 |  |
| RES | C20ORF3 | 12 | 145698 | 3,49E-03 |  |
| RES | C6ORF153 | 11 | 145698 | 5,59E-03 |  |
| RES | C7ORF50 | 23 | 145698 | 1,95E-05 | (*) |
| RES | CBLC | 11 | 145698 | 5,59E-03 |  |
| RES | CBS | 14 | 145698 | 1,36E-03 |  |
| RES | CCDC124 | 17 | 145698 | 3,30E-04 |  |
| RES | CCM2 | 10 | 145698 | 8,95E-03 |  |
| RES | CCNB1IP1 | 23 | 145698 | 1,95E-05 | (*) |
| RES | CCNB2 | 15 | 145698 | 8,47E-04 |  |
| RES | CDK4 | 39 | 145698 | 9,89E-09 | (*) |
| RES | CHD8 | 10 | 145698 | 8,95E-03 |  |
| RES | CHID1 | 14 | 145698 | 1,36E-03 |  |
| RES | CHPF | 11 | 145698 | 5,59E-03 |  |
| RES | CLEC11A | 10 | 145698 | 8,95E-03 |  |
| RES | CLPP | 18 | 145698 | 2,06E-04 |  |
| RES | COBRA1 | 15 | 145698 | 8,47E-04 |  |
| RES | COQ5 | 10 | 145698 | 8,95E-03 |  |
| RES | CREG1 | 19 | 145698 | 1,28E-04 |  |
| RES | CRMP1 | 10 | 145698 | 8,95E-03 |  |
| RES | CSTF2 | 11 | 145698 | 5,59E-03 |  |
| RES | CXCL5 | 12 | 145698 | 3,49E-03 |  |
| RES | CXORF40B | 12 | 145698 | 3,49E-03 |  |
| RES | DCTN3 | 12 | 145698 | 3,49E-03 |  |
| RES | DCUN1D5 | 13 | 145698 | 2,18E-03 |  |
| RES | DCXR | 14 | 145698 | 1,36E-03 |  |
| RES | DDA1 | 13 | 145698 | 2,18E-03 |  |
| RES | DDX11 | 12 | 145698 | 3,49E-03 |  |
| RES | EDF1 | 14 | 145698 | 1,36E-03 |  |
| RES | EEF1A2 | 30 | 145698 | 7,17E-07 | (*) |
| RES | EFHD2 | 12 | 145698 | 3,49E-03 |  |
| RES | ENO2 | 11 | 145698 | 5,59E-03 |  |
| RES | ESRRA | 20 | 145698 | 8,01E-05 |  |
| RES | EXOSC5 | 19 | 145698 | 1,28E-04 |  |
| RES | FAM125A | 13 | 145698 | 2,18E-03 |  |
| RES | FAM60A | 10 | 145698 | 8,95E-03 |  |
| RES | FAM83A | 10 | 145698 | 8,95E-03 |  |
| RES | FEN1 | 25 | 145698 | 7,58E-06 | (*) |
| RES | FGFR1 | 13 | 145698 | 2,18E-03 |  |
| RES | FKBP4 | 20 | 145698 | 8,01E-05 |  |
| RES | FMNL1 | 13 | 145698 | 2,18E-03 |  |
| RES | FOXA2 | 10 | 145698 | 8,95E-03 |  |
| RES | FOXP4 | 13 | 145698 | 2,18E-03 |  |
| RES | FSCN1 | 17 | 145698 | 3,30E-04 |  |
| RES | G0S2 | 17 | 145698 | 3,30E-04 |  |
| RES | G6PC3 | 14 | 145698 | 1,36E-03 |  |
| RES | GADD45GIP1 | 10 | 145698 | 8,95E-03 |  |
| RES | GDF15 | 44 | 145698 | 1,17E-09 | (*) |
| RES | GFPT2 | 16 | 145698 | 5,29E-04 |  |
| RES | GINS2 | 11 | 145698 | 5,59E-03 |  |
| RES | GIT1 | 16 | 145698 | 5,29E-04 |  |
| RES | GPKOW | 14 | 145698 | 1,36E-03 |  |
| RES | GPX2 | 24 | 145698 | 1,21E-05 | (*) |
| RES | GSTM3 | 12 | 145698 | 3,49E-03 |  |
| RES | GSTZ1 | 11 | 145698 | 5,59E-03 |  |
| RES | GTF3C1 | 21 | 145698 | 5,00E-05 |  |
| RES | H2AFV | 11 | 145698 | 5,59E-03 |  |
| RES | HGS | 11 | 145698 | 5,59E-03 |  |
| RES | HMG20B | 10 | 145698 | 8,95E-03 |  |
| RES | HTR3A | 11 | 145698 | 5,59E-03 |  |
| RES | IER3 | 18 | 145698 | 2,06E-04 |  |
| RES | IKBKG | 11 | 145698 | 5,59E-03 |  |
| RES | INTS1 | 13 | 145698 | 2,18E-03 |  |
| RES | IRAK1 | 44 | 145698 | 1,17E-09 | (*) |
| RES | ITFG3 | 10 | 145698 | 8,95E-03 |  |
| RES | ITPK1 | 15 | 145698 | 8,47E-04 |  |
| RES | JUP | 10 | 145698 | 8,95E-03 |  |
| RES | KAT2A | 11 | 145698 | 5,59E-03 |  |
| RES | KATNB1 | 13 | 145698 | 2,18E-03 |  |
| RES | KCNE4 | 10 | 145698 | 8,95E-03 |  |
| RES | KIAA0999 | 24 | 145698 | 1,21E-05 | (*) |
| RES | KIAA1191 | 15 | 145698 | 8,47E-04 |  |
| RES | KRT5 | 22 | 145698 | 3,12E-05 | (*) |
| RES | KRT81 | 31 | 145698 | 4,47E-07 | (*) |
| RES | KRT83 | 29 | 145698 | 1,15E-06 | (*) |
| RES | KRT86 | 13 | 145698 | 2,18E-03 |  |
| RES | LAMP1 | 15 | 145698 | 8,47E-04 |  |
| RES | LASS2 | 13 | 145698 | 2,18E-03 |  |
| RES | LSM4 | 21 | 145698 | 5,00E-05 |  |
| RES | LUC7L | 11 | 145698 | 5,59E-03 |  |
| RES | MAP2K2 | 30 | 145698 | 7,17E-07 | (*) |
| RES | MAP3K7IP1 | 10 | 145698 | 8,95E-03 |  |
| RES | MAZ | 19 | 145698 | 1,28E-04 |  |
| RES | MGAT4B | 93 | 145698 | 0,00E+00 | (*) |
| RES | MLF2 | 32 | 145698 | 2,79E-07 | (*) |
| RES | MRPL12 | 15 | 145698 | 8,47E-04 |  |
| RES | MRPL24 | 13 | 145698 | 2,18E-03 |  |
| RES | MRPL38 | 26 | 145698 | 4,73E-06 | (*) |
| RES | MRPL52 | 12 | 145698 | 3,49E-03 |  |
| RES | MRPS11 | 10 | 145698 | 8,95E-03 |  |
| RES | MRPS2 | 17 | 145698 | 3,30E-04 |  |
| RES | MRPS26 | 10 | 145698 | 8,95E-03 |  |
| RES | MRPS34 | 19 | 145698 | 1,28E-04 |  |
| RES | MTHFD2 | 14 | 145698 | 1,36E-03 |  |
| RES | MXD4 | 10 | 145698 | 8,95E-03 |  |
| RES | MYBL2 | 21 | 145698 | 5,00E-05 |  |
| RES | MYO19 | 19 | 145698 | 1,28E-04 |  |
| RES | NCAPD2 | 14 | 145698 | 1,36E-03 |  |
| RES | NCAPH2 | 18 | 145698 | 2,06E-04 |  |
| RES | NDUFAB1 | 20 | 145698 | 8,01E-05 |  |
| RES | NDUFAF3 | 13 | 145698 | 2,18E-03 |  |
| RES | NDUFS3 | 16 | 145698 | 5,29E-04 |  |
| RES | NELF | 13 | 145698 | 2,18E-03 |  |
| RES | NLE1 | 11 | 145698 | 5,59E-03 |  |
| RES | NME4 | 14 | 145698 | 1,36E-03 |  |
| RES | NOC4L | 12 | 145698 | 3,49E-03 |  |
| RES | NOL4 | 11 | 145698 | 5,59E-03 |  |
| RES | NOMO1 | 11 | 145698 | 5,59E-03 |  |
| RES | NR2F6 | 13 | 145698 | 2,18E-03 |  |
| RES | NSFL1C | 19 | 145698 | 1,28E-04 |  |
| RES | NTHL1 | 14 | 145698 | 1,36E-03 |  |
| RES | OGFR | 10 | 145698 | 8,95E-03 |  |
| RES | PDIA4 | 29 | 145698 | 1,15E-06 | (*) |
| RES | PDLIM7 | 13 | 145698 | 2,18E-03 |  |
| RES | PGLS | 10 | 145698 | 8,95E-03 |  |
| RES | PIR | 16 | 145698 | 5,29E-04 |  |
| RES | PKN3 | 10 | 145698 | 8,95E-03 |  |
| RES | PLCD3 | 14 | 145698 | 1,36E-03 |  |
| RES | PLEKHJ1 | 10 | 145698 | 8,95E-03 |  |
| RES | PLK1 | 17 | 145698 | 3,30E-04 |  |
| RES | PNPLA2 | 14 | 145698 | 1,36E-03 |  |
| RES | PPP2R5C | 10 | 145698 | 8,95E-03 |  |
| RES | PRC1 | 11 | 145698 | 5,59E-03 |  |
| RES | PRKCDBP | 12 | 145698 | 3,49E-03 |  |
| RES | PRR13 | 15 | 145698 | 8,47E-04 |  |
| RES | PSMC3IP | 13 | 145698 | 2,18E-03 |  |
| RES | PTBP1 | 19 | 145698 | 1,28E-04 |  |
| RES | PTGES2 | 18 | 145698 | 2,06E-04 |  |
| RES | RAB1B | 17 | 145698 | 3,30E-04 |  |
| RES | RAD23A | 15 | 145698 | 8,47E-04 |  |
| RES | RBCK1 | 23 | 145698 | 1,95E-05 | (*) |
| RES | RBM23 | 13 | 145698 | 2,18E-03 |  |
| RES | RFXANK | 16 | 145698 | 5,29E-04 |  |
| RES | RHBDD2 | 10 | 145698 | 8,95E-03 |  |
| RES | RMND5B | 10 | 145698 | 8,95E-03 |  |
| RES | RNASEH2A | 24 | 145698 | 1,21E-05 | (*) |
| RES | RPUSD1 | 10 | 145698 | 8,95E-03 |  |
| RES | S100A9 | 30 | 145698 | 7,17E-07 | (*) |
| RES | SBF1 | 13 | 145698 | 2,18E-03 |  |
| RES | SCRIB | 15 | 145698 | 8,47E-04 |  |
| RES | SEC24C | 13 | 145698 | 2,18E-03 |  |
| RES | SF3A1 | 13 | 145698 | 2,18E-03 |  |
| RES | SGTA | 17 | 145698 | 3,30E-04 |  |
| RES | SHARPIN | 12 | 145698 | 3,49E-03 |  |
| RES | SIGMAR1 | 15 | 145698 | 8,47E-04 |  |
| RES | SLC1A5 | 21 | 145698 | 5,00E-05 |  |
| RES | SLC25A1 | 14 | 145698 | 1,36E-03 |  |
| RES | SLC25A19 | 13 | 145698 | 2,18E-03 |  |
| RES | SNAPIN | 14 | 145698 | 1,36E-03 |  |
| RES | SPAG5 | 19 | 145698 | 1,28E-04 |  |
| RES | SPP1 | 49 | 145698 | 3,25E-10 | (*) |
| RES | SRCAP | 16 | 145698 | 5,29E-04 |  |
| RES | SYNGR2 | 16 | 145698 | 5,29E-04 |  |
| RES | T | 29 | 145698 | 1,15E-06 | (*) |
| RES | TACC3 | 20 | 145698 | 8,01E-05 |  |
| RES | TBCD | 18 | 145698 | 2,06E-04 |  |
| RES | TCF25 | 13 | 145698 | 2,18E-03 |  |
| RES | TESC | 16 | 145698 | 5,29E-04 |  |
| RES | THOC6 | 10 | 145698 | 8,95E-03 |  |
| RES | TIMELESS | 15 | 145698 | 8,47E-04 |  |
| RES | TIMM44 | 13 | 145698 | 2,18E-03 |  |
| RES | TIMM50 | 11 | 145698 | 5,59E-03 |  |
| RES | TK1 | 15 | 145698 | 8,47E-04 |  |
| RES | TMEM183A | 10 | 145698 | 8,95E-03 |  |
| RES | TNFRSF12A | 18 | 145698 | 2,06E-04 |  |
| RES | TPD52L1 | 17 | 145698 | 3,30E-04 |  |
| RES | TRAF7 | 26 | 145698 | 4,73E-06 | (*) |
| RES | TRIB3 | 24 | 145698 | 1,21E-05 | (*) |
| RES | TRIM16L | 23 | 145698 | 1,95E-05 | (*) |
| RES | TSFM | 12 | 145698 | 3,49E-03 |  |
| RES | TUBB2A | 15 | 145698 | 8,47E-04 |  |
| RES | TUBB3 | 42 | 145698 | 2,16E-09 | (*) |
| RES | TXNL4A | 14 | 145698 | 1,36E-03 |  |
| RES | UBE2S | 18 | 145698 | 2,06E-04 |  |
| RES | ULK1 | 11 | 145698 | 5,59E-03 |  |
| RES | VAC14 | 10 | 145698 | 8,95E-03 |  |
| RES | VPS72 | 16 | 145698 | 5,29E-04 |  |
| RES | VTI1B | 10 | 145698 | 8,95E-03 |  |
| RES | WBSCR16 | 10 | 145698 | 8,95E-03 |  |
| RES | WDR18 | 26 | 145698 | 4,73E-06 | (*) |
| RES | WDR74 | 15 | 145698 | 8,47E-04 |  |
| RES | XBP1 | 17 | 145698 | 3,30E-04 |  |
| RES | YDJC | 10 | 145698 | 8,95E-03 |  |
| RES | YIF1B | 11 | 145698 | 5,59E-03 |  |
| RES | ZAK | 16 | 145698 | 5,29E-04 |  |
| RES | ZFPL1 | 10 | 145698 | 8,95E-03 |  |
| STO | ABCF1 | 40 | 81606 | 1,15E-06 | (*) |
| STO | ACSL5 | 16 | 81606 | 4,21E-03 |  |
| STO | ALG13 | 21 | 81606 | 7,63E-04 |  |
| STO | ANKRD9 | 26 | 81606 | 1,38E-04 |  |
| STO | ATP1B1 | 16 | 81606 | 4,21E-03 |  |
| STO | BRD2 | 25 | 81606 | 1,94E-04 |  |
| STO | BTBD1 | 26 | 81606 | 1,38E-04 |  |
| STO | C9ORF114 | 14 | 81606 | 8,35E-03 |  |
| STO | CCNB1IP1 | 15 | 81606 | 5,93E-03 |  |
| STO | CGA | 17 | 81606 | 2,99E-03 |  |
| STO | CR1L | 21 | 81606 | 7,63E-04 |  |
| STO | CTSH | 26 | 81606 | 1,38E-04 |  |
| STO | DDX41 | 21 | 81606 | 7,63E-04 |  |
| STO | EIF2A | 30 | 81606 | 3,52E-05 | (*) |
| STO | EIF3E | 15 | 81606 | 5,93E-03 |  |
| STO | ERCC3 | 15 | 81606 | 5,93E-03 |  |
| STO | FAM55C | 14 | 81606 | 8,35E-03 |  |
| STO | FBN1 | 21 | 81606 | 7,63E-04 |  |
| STO | FBXL6 | 16 | 81606 | 4,21E-03 |  |
| STO | FKBP4 | 30 | 81606 | 3,52E-05 | (*) |
| STO | FLNA | 15 | 81606 | 5,93E-03 |  |
| STO | FOSL1 | 14 | 81606 | 8,35E-03 |  |
| STO | GBA2 | 16 | 81606 | 4,21E-03 |  |
| STO | GLTSCR2 | 35 | 81606 | 6,36E-06 | (*) |
| STO | GTPBP4 | 14 | 81606 | 8,35E-03 |  |
| STO | HDAC1 | 182 | 81606 | 1,43E-10 | (*) |
| STO | HSPA4 | 40 | 81606 | 1,15E-06 | (*) |
| STO | IARS | 33 | 81606 | 1,26E-05 | (*) |
| STO | KDM5C | 19 | 81606 | 1,51E-03 |  |
| STO | KIFC3 | 16 | 81606 | 4,21E-03 |  |
| STO | KLK6 | 15 | 81606 | 5,93E-03 |  |
| STO | KREMEN2 | 19 | 81606 | 1,51E-03 |  |
| STO | LOC81691 | 16 | 81606 | 4,21E-03 |  |
| STO | MEIS3 | 18 | 81606 | 2,13E-03 |  |
| STO | NFYC | 29 | 81606 | 4,95E-05 |  |
| STO | PDIA4 | 25 | 81606 | 1,94E-04 |  |
| STO | PGAM1 | 22 | 81606 | 5,42E-04 |  |
| STO | PLSCR1 | 31 | 81606 | 2,50E-05 | (*) |
| STO | PTGFRN | 289 | 81606 | 0,00E+00 | (*) |
| STO | PTK2B | 40 | 81606 | 1,15E-06 | (*) |
| STO | QSOX1 | 26 | 81606 | 1,38E-04 |  |
| STO | RBMS1 | 43 | 81606 | 4,13E-07 | (*) |
| STO | RRM2 | 50 | 81606 | 3,79E-08 | (*) |
| STO | SLMO2 | 16 | 81606 | 4,21E-03 |  |
| STO | TACC2 | 21 | 81606 | 7,63E-04 |  |
| STO | TBC1D8 | 25 | 81606 | 1,94E-04 |  |
| STO | THBS3 | 15 | 81606 | 5,93E-03 |  |
| STO | WDR45L | 16 | 81606 | 4,21E-03 |  |
| TES | ABCB7 | 18 | 112913 | 9,88E-06 | (*) |
| TES | ABCD3 | 23 | 112913 | 4,02E-07 | (*) |
| TES | ADAM10 | 15 | 112913 | 6,75E-05 |  |
| TES | AIF1L | 15 | 112913 | 6,75E-05 |  |
| TES | AMOTL2 | 37 | 112913 | 2,53E-10 | (*) |
| TES | ANKRD17 | 10 | 112913 | 1,66E-03 |  |
| TES | ANP32E | 10 | 112913 | 1,66E-03 |  |
| TES | ARHGDIB | 27 | 112913 | 3,12E-08 | (*) |
| TES | ARID1A | 10 | 112913 | 1,66E-03 |  |
| TES | ARMCX2 | 10 | 112913 | 1,66E-03 |  |
| TES | ATCAY | 10 | 112913 | 1,66E-03 |  |
| TES | ATXN2 | 11 | 112913 | 8,74E-04 |  |
| TES | AURKB | 16 | 112913 | 3,56E-05 | (*) |
| TES | BCAT1 | 18 | 112913 | 9,88E-06 | (*) |
| TES | BMP1 | 17 | 112913 | 1,87E-05 | (*) |
| TES | C10ORF26 | 10 | 112913 | 1,66E-03 |  |
| TES | C12ORF23 | 13 | 112913 | 2,43E-04 |  |
| TES | C14ORF147 | 19 | 112913 | 5,21E-06 | (*) |
| TES | C7ORF44 | 12 | 112913 | 4,61E-04 |  |
| TES | CAMKV | 33 | 112913 | 4,59E-10 | (*) |
| TES | CD276 | 53 | 112913 | 2,33E-10 | (*) |
| TES | CDCA3 | 10 | 112913 | 1,66E-03 |  |
| TES | CDCA7 | 18 | 112913 | 9,88E-06 | (*) |
| TES | CDH6 | 16 | 112913 | 3,56E-05 | (*) |
| TES | CENPK | 12 | 112913 | 4,61E-04 |  |
| TES | CGGBP1 | 12 | 112913 | 4,61E-04 |  |
| TES | CLDN12 | 13 | 112913 | 2,43E-04 |  |
| TES | CPXM1 | 12 | 112913 | 4,61E-04 |  |
| TES | CRABP2 | 15 | 112913 | 6,75E-05 |  |
| TES | DDHD2 | 10 | 112913 | 1,66E-03 |  |
| TES | DEF8 | 77 | 112913 | 4,33E-13 | (*) |
| TES | DEK | 11 | 112913 | 8,74E-04 |  |
| TES | DERA | 11 | 112913 | 8,74E-04 |  |
| TES | DPH3 | 10 | 112913 | 1,66E-03 |  |
| TES | DPPA4 | 21 | 112913 | 1,45E-06 | (*) |
| TES | DSCR3 | 11 | 112913 | 8,74E-04 |  |
| TES | EHD2 | 12 | 112913 | 4,61E-04 |  |
| TES | EIF2S3 | 32 | 112913 | 1,28E-09 | (*) |
| TES | ENC1 | 15 | 112913 | 6,75E-05 |  |
| TES | ENO2 | 46 | 112913 | 5,14E-10 | (*) |
| TES | EXTL2 | 17 | 112913 | 1,87E-05 | (*) |
| TES | FADS1 | 29 | 112913 | 8,57E-09 | (*) |
| TES | FAM115A | 22 | 112913 | 7,63E-07 | (*) |
| TES | FAM60A | 85 | 112913 | 3,00E-10 | (*) |
| TES | FAM70A | 23 | 112913 | 4,02E-07 | (*) |
| TES | FPGT | 11 | 112913 | 8,74E-04 |  |
| TES | FRMD4B | 15 | 112913 | 6,75E-05 |  |
| TES | FZD6 | 18 | 112913 | 9,88E-06 | (*) |
| TES | GAP43 | 11 | 112913 | 8,74E-04 |  |
| TES | GJA1 | 34 | 112913 | 2,11E-10 | (*) |
| TES | GPR162 | 11 | 112913 | 8,74E-04 |  |
| TES | GPRASP2 | 13 | 112913 | 2,43E-04 |  |
| TES | GPX8 | 12 | 112913 | 4,61E-04 |  |
| TES | GRB2 | 18 | 112913 | 9,88E-06 | (*) |
| TES | GTPBP5 | 16 | 112913 | 3,56E-05 | (*) |
| TES | GTSE1 | 16 | 112913 | 3,56E-05 | (*) |
| TES | GYS1 | 16 | 112913 | 3,56E-05 | (*) |
| TES | HOXA1 | 11 | 112913 | 8,74E-04 |  |
| TES | HOXB3 | 12 | 112913 | 4,61E-04 |  |
| TES | HSPE1 | 17 | 112913 | 1,87E-05 | (*) |
| TES | IFI6 | 11 | 112913 | 8,74E-04 |  |
| TES | IREB2 | 14 | 112913 | 1,28E-04 |  |
| TES | ITIH5 | 13 | 112913 | 2,43E-04 |  |
| TES | KCTD21 | 10 | 112913 | 1,66E-03 |  |
| TES | KIAA1033 | 10 | 112913 | 1,66E-03 |  |
| TES | KIF20A | 12 | 112913 | 4,61E-04 |  |
| TES | KIF4A | 12 | 112913 | 4,61E-04 |  |
| TES | KLHL13 | 23 | 112913 | 4,02E-07 | (*) |
| TES | KRT18 | 10 | 112913 | 1,66E-03 |  |
| TES | LARS2 | 12 | 112913 | 4,61E-04 |  |
| TES | LASP1 | 23 | 112913 | 4,02E-07 | (*) |
| TES | LBH | 14 | 112913 | 1,28E-04 |  |
| TES | LEPROTL1 | 12 | 112913 | 4,61E-04 |  |
| TES | LOXL2 | 26 | 112913 | 5,93E-08 | (*) |
| TES | LRMP | 16 | 112913 | 3,56E-05 | (*) |
| TES | MAGOHB | 16 | 112913 | 3,56E-05 | (*) |
| TES | MAPRE2 | 12 | 112913 | 4,61E-04 |  |
| TES | MCFD2 | 34 | 112913 | 2,11E-10 | (*) |
| TES | MCM2 | 49 | 112913 | 1,20E-11 | (*) |
| TES | MDK | 16 | 112913 | 3,56E-05 | (*) |
| TES | MED22 | 14 | 112913 | 1,28E-04 |  |
| TES | MEIS2 | 27 | 112913 | 3,12E-08 | (*) |
| TES | MID1 | 24 | 112913 | 2,12E-07 | (*) |
| TES | MKI67IP | 22 | 112913 | 7,63E-07 | (*) |
| TES | MLLT11 | 17 | 112913 | 1,87E-05 | (*) |
| TES | MOBKL3 | 11 | 112913 | 8,74E-04 |  |
| TES | NAT11 | 20 | 112913 | 2,75E-06 | (*) |
| TES | NAT13 | 24 | 112913 | 2,12E-07 | (*) |
| TES | NECAP1 | 20 | 112913 | 2,75E-06 | (*) |
| TES | NHLH2 | 15 | 112913 | 6,75E-05 |  |
| TES | NTRK2 | 25 | 112913 | 1,12E-07 | (*) |
| TES | NUDT5 | 10 | 112913 | 1,66E-03 |  |
| TES | OSBPL5 | 10 | 112913 | 1,66E-03 |  |
| TES | PAM | 23 | 112913 | 4,02E-07 | (*) |
| TES | PBX3 | 11 | 112913 | 8,74E-04 |  |
| TES | PCSK9 | 10 | 112913 | 1,66E-03 |  |
| TES | PCYOX1 | 12 | 112913 | 4,61E-04 |  |
| TES | PDHA1 | 19 | 112913 | 5,21E-06 | (*) |
| TES | PDPN | 49 | 112913 | 1,20E-11 | (*) |
| TES | PFN1 | 12 | 112913 | 4,61E-04 |  |
| TES | PGAM1 | 10 | 112913 | 1,66E-03 |  |
| TES | PHF6 | 11 | 112913 | 8,74E-04 |  |
| TES | PRDX2 | 11 | 112913 | 8,74E-04 |  |
| TES | PSAT1 | 16 | 112913 | 3,56E-05 | (*) |
| TES | PTPRO | 10 | 112913 | 1,66E-03 |  |
| TES | PXDN | 13 | 112913 | 2,43E-04 |  |
| TES | RAB3GAP1 | 12 | 112913 | 4,61E-04 |  |
| TES | RAB6A | 10 | 112913 | 1,66E-03 |  |
| TES | RCAN2 | 13 | 112913 | 2,43E-04 |  |
| TES | REEP5 | 11 | 112913 | 8,74E-04 |  |
| TES | RFC4 | 13 | 112913 | 2,43E-04 |  |
| TES | RHEB | 12 | 112913 | 4,61E-04 |  |
| TES | RNF185 | 15 | 112913 | 6,75E-05 |  |
| TES | RPS10 | 10 | 112913 | 1,66E-03 |  |
| TES | RPS2 | 28 | 112913 | 1,66E-08 | (*) |
| TES | SCRN1 | 10 | 112913 | 1,66E-03 |  |
| TES | SCYL2 | 10 | 112913 | 1,66E-03 |  |
| TES | SETD4 | 10 | 112913 | 1,66E-03 |  |
| TES | SH3BP5L | 20 | 112913 | 2,75E-06 | (*) |
| TES | SLC1A3 | 19 | 112913 | 5,21E-06 | (*) |
| TES | SLC25A13 | 10 | 112913 | 1,66E-03 |  |
| TES | SLC2A1 | 125 | 112913 | 0,00E+00 | (*) |
| TES | SLC35B4 | 12 | 112913 | 4,61E-04 |  |
| TES | SLC38A1 | 18 | 112913 | 9,88E-06 | (*) |
| TES | SLC38A2 | 32 | 112913 | 1,28E-09 | (*) |
| TES | SLC7A3 | 13 | 112913 | 2,43E-04 |  |
| TES | SNAP25 | 10 | 112913 | 1,66E-03 |  |
| TES | SNCAIP | 12 | 112913 | 4,61E-04 |  |
| TES | STK40 | 10 | 112913 | 1,66E-03 |  |
| TES | SV2A | 37 | 112913 | 2,53E-10 | (*) |
| TES | SYT4 | 26 | 112913 | 5,93E-08 | (*) |
| TES | TBC1D13 | 12 | 112913 | 4,61E-04 |  |
| TES | TCOF1 | 10 | 112913 | 1,66E-03 |  |
| TES | TMED5 | 11 | 112913 | 8,74E-04 |  |
| TES | TMEM126B | 10 | 112913 | 1,66E-03 |  |
| TES | TMEM184B | 26 | 112913 | 5,93E-08 | (*) |
| TES | TMEM2 | 10 | 112913 | 1,66E-03 |  |
| TES | TMEM50A | 32 | 112913 | 1,28E-09 | (*) |
| TES | TNFRSF10B | 55 | 112913 | 2,29E-10 | (*) |
| TES | TNFRSF19 | 10 | 112913 | 1,66E-03 |  |
| TES | TPBG | 11 | 112913 | 8,74E-04 |  |
| TES | TUBB2B | 51 | 112913 | 3,70E-10 | (*) |
| TES | TYW1 | 11 | 112913 | 8,74E-04 |  |
| TES | UBE2G2 | 10 | 112913 | 1,66E-03 |  |
| TES | URG4 | 14 | 112913 | 1,28E-04 |  |
| TES | USP33 | 15 | 112913 | 6,75E-05 |  |
| TES | VGLL4 | 16 | 112913 | 3,56E-05 | (*) |
| TES | VTA1 | 12 | 112913 | 4,61E-04 |  |
| TES | WDR75 | 17 | 112913 | 1,87E-05 | (*) |
| TES | XPC | 13 | 112913 | 2,43E-04 |  |
| TES | ZC4H2 | 29 | 112913 | 8,57E-09 | (*) |
| TES | ZMPSTE24 | 12 | 112913 | 4,61E-04 |  |
| TES | ZNF146 | 12 | 112913 | 4,61E-04 |  |
| TES | ZNF212 | 16 | 112913 | 3,56E-05 | (*) |
| TES | ZNF317 | 11 | 112913 | 8,74E-04 |  |
| TES | ZNF512 | 27 | 112913 | 3,12E-08 | (*) |
