# Supplementary material for: Identification of tumor-associated cassette exons in human cancer through EST-based computational prediction and experimental validation
Source: Mol Cancer. 2010 Sep 2;9:230. doi: 10.1186/1476-4598-9-230 (PMC2941758; doi:10.1186/1476-4598-9-230)
Supplement: Additional file 3 — Top 10 represented GO terms among genes over-represented in tumor (T/TT) and normal (N/NN) tissues. For each GO term this table reports the GO identifier (GO_Id), the number of genes, the P value (P-val) and the description (GO_Description). Only Gene Ontology terms assigned to more than 50 human genes were considered. [file 1476-4598-9-230-S3.DOC]

| **T/TT genes** |  |  |  |
| --- | --- | --- | --- |
| **GO_Id** | **# Genes** | **P-val** | **GO_Description** |
| GO:0048522 | 286 | 4.99E-04 | positive regulation of cellular process |
| GO:0050793 | 226 | 8.54E-05 | regulation of developmental process |
| GO:0042127 | 156 | 6.44E-04 | regulation of cell proliferation |
| GO:0019725 | 95 | 7.32E-03 | cellular homeostasis |
| GO:0051093 | 95 | 4.61E-06 | negative regulation of developmental process |
| GO:0009889 | 83 | 4.35E-05 | regulation of biosynthetic process |
| GO:0050789 | 71 | 5.79E-06 | regulation of biological process |
| GO:0006916 | 63 | 9.94E-07 | anti-apoptosis |
| GO:0040008 | 54 | 1.08E-03 | regulation of growth |
| GO:0007346 | 37 | 5.85E-04 | regulation of mitotic cell cycle |
|  |  |  |  |
| **N/NN genes** |  |  |  |
| **GO_Id** | **# Genes** | **P-val** | **GO_Description** |
| GO:0006468 | 289 | 3.25E-04 | protein amino acid phosphorylation |
| GO:0032879 | 193 | 4.17E-03 | regulation of localization |
| GO:0051056 | 130 | 5.71E-03 | regulation of small GTPase mediated signal transduction |
| GO:0010324 | 118 | 1.49E-07 | membrane invagination |
| GO:0006897 | 118 | 1.49E-07 | Endocytosis |
| GO:0009725 | 89 | 7.30E-03 | response to hormone stimulus |
| GO:0019637 | 85 | 8.93E-04 | organophosphate metabolic process |
| GO:0006644 | 85 | 8.93E-04 | phospholipid metabolic process |
| GO:0046486 | 74 | 1.28E-03 | glycerolipid metabolic process |
| GO:0032940 | 71 | 1.06E-03 | secretion by cell |
