# Supplementary material for: Identification of tumor-associated cassette exons in human cancer through EST-based computational prediction and experimental validation
Source: Mol Cancer. 2010 Sep 2;9:230. doi: 10.1186/1476-4598-9-230 (PMC2941758; doi:10.1186/1476-4598-9-230)
Supplement: Additional file 4 — Genes and splicing sites showing a normal or tumor specific expression. For each tissue this table reports the number of genes and splicing sites with a labeled expression pattern as described in the text (total numbers after the Bonferroni correction at the 0.05 confidence level are in the brackets) [file 1476-4598-9-230-S4.DOC]

| **Tissue** | **Number of Genes** | | | **Number of Splice Sites** | | **Total Genes** | **Total Splice Sites** |
| --- | --- | --- | --- | --- | --- | --- | --- |
|  | **N** | **T** | **E** | **N or NN** | **T or TT** |  |  |
| BMA | 68 (47) | 203 (161) | 120 | 191 (150) | 476 (397) | 391 | 667 |
| BRE | 128 (85) | 683 (605) | 689 | 495 (441) | 1368 (1178) | 1500 | 1863 |
| CNS | 2488 (1861) | 1598 (1025) | 3472 | 6851 (5290) | 11585 (9735) | 7558 | 18436 |
| COL | 269 (186) | 246 (222) | 1003 | 1252 (1023) | 423 (379) | 1518 | 1675 |
| DER | 391 (271) | 1064 (877) | 1223 | 1629 (1394) | 4644 (4009) | 2678 | 6273 |
| END | 524 (434) | 350 (299) | 1038 | 1680 (1415) | 1427 (1311) | 1912 | 3107 |
| EYE | 324 (277) | 720 (501) | 894 | 212 (185) | 4540 (3756) | 1938 | 4752 |
| INT | 213 (157) | 161 (125) | 410 | 627 (545) | 590 (536) | 784 | 1217 |
| KID | 581 (468) | 442 (318) | 961 | 1843 (1567) | 2044 (1727) | 1984 | 3887 |
| LIV | 210 (157) | 1102 (769) | 825 | 741 (609) | 5285 (4439) | 2137 | 6026 |
| LYM | 583 (472) | 623 (454) | 897 | 1385 (1223) | 4052 (3455) | 2103 | 5437 |
| MSK | 572 (426) | 560 (404) | 796 | 1784 (1445) | 2980 (2488) | 1928 | 4764 |
| PLA | 401 (335) | 753 (454) | 884 | 442 (390) | 4916 (3952) | 2038 | 5358 |
| PRO | 527 (311) | 132 (122) | 737 | 2407 (1821) | 400 (365) | 1396 | 2807 |
| RES | 502 (324) | 799 (664) | 915 | 1832 (1415) | 3307 (2859) | 2216 | 5139 |
| STO | 196 (143) | 195 (179) | 424 | 837 (713) | 369 (352) | 815 | 1206 |
| TES | 707 (501) | 950 (710) | 1005 | 2175 (1813) | 3744 (3182) | 2662 | 5919 |
