# Supplementary figures and images for: Identification of tumor-associated cassette exons in human cancer through EST-based computational prediction and experimental validation
Source: Mol Cancer. 2010 Sep 2;9:230. doi: 10.1186/1476-4598-9-230 (PMC2941758; doi:10.1186/1476-4598-9-230)

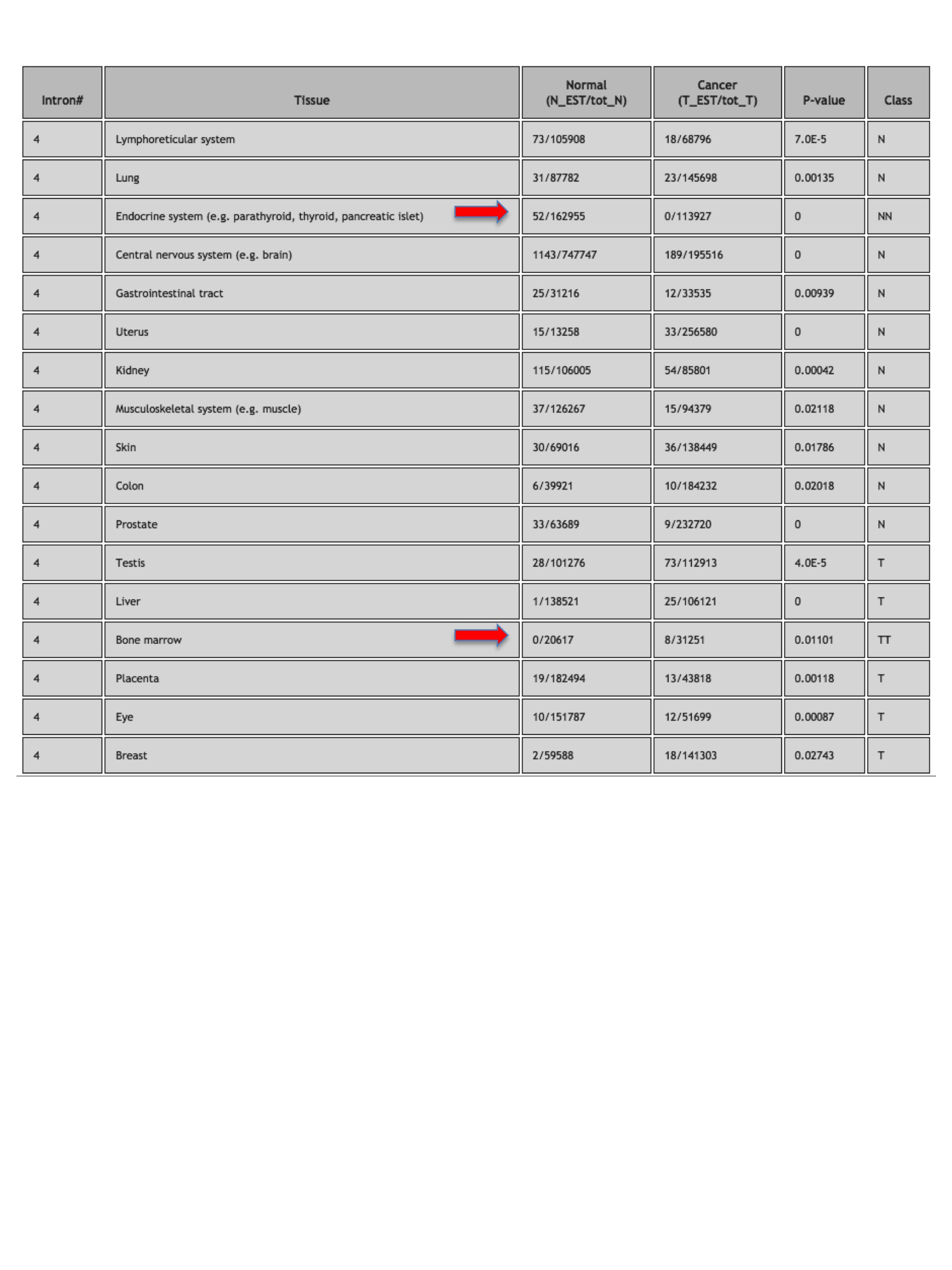

Supplement: Additional file 5 — Heterogeneous classification of the first intron of the PPP2R1A gene (intron #4 following ASPidDB nomenclature located at chr19:57385240-57397008). Expression pattern of intron #4 of the PPP2R1A gene where is shown the number of supporting ESTs from normal and cancer tissues, the statistical significance and the classification. This intron is exclusively expressed in tumor bone marrow (BMA) and in normal endocrine system (END) (see red arrows). [file 1476-4598-9-230-S5.JPEG]

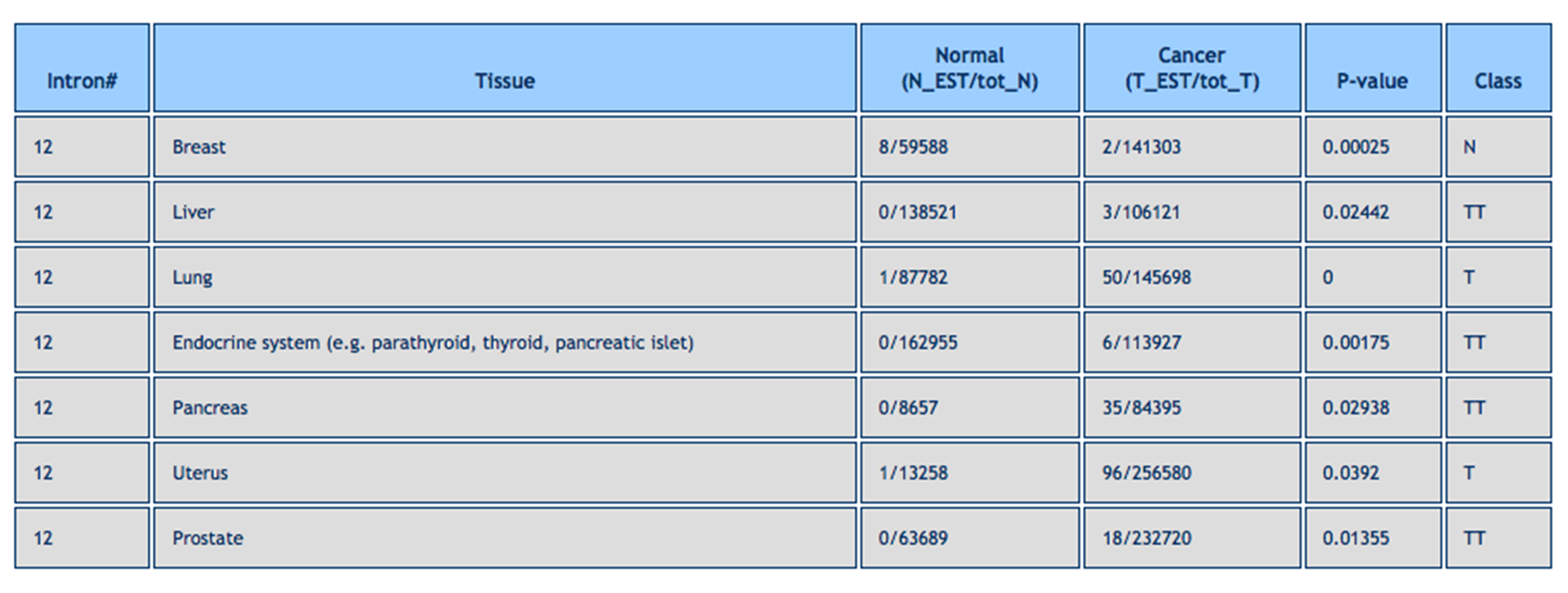

Supplement: Additional file 9 — Expression pattern of intron #12 of KRT7 gene. ASPicDB table relative to the expression pattern of intron #12 of the KRT7 gene (following ASPicDB nomenclature, located at chr12:50917621-50918730) showing the number of supporting ESTs from normal and cancer tissues, the statistical significance and the classification. [file 1476-4598-9-230-S9.TIFF]

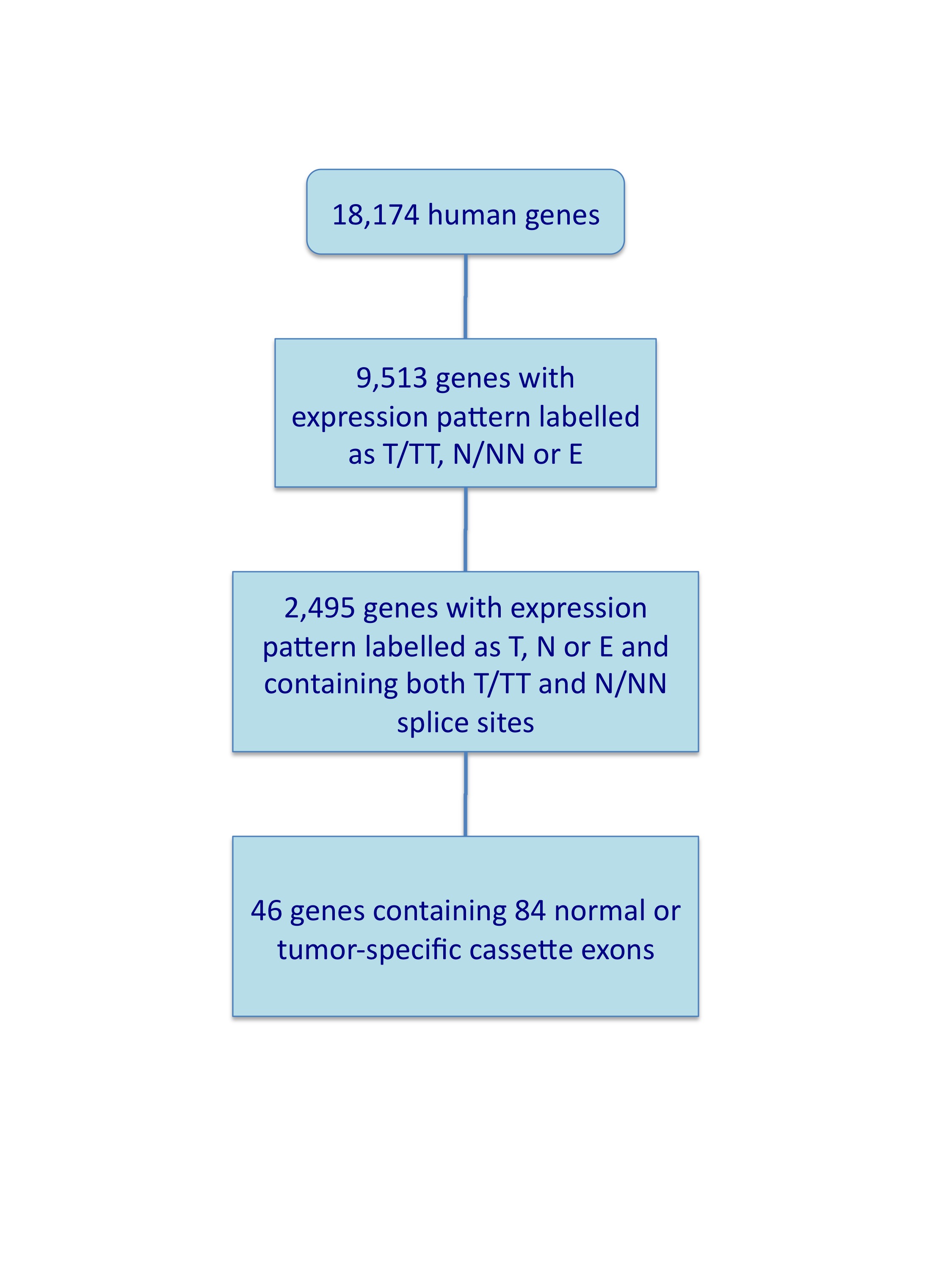

Supplement: Additional file 10 — Data flux flowchart. The flowchart reports the number of genes at each step in the detection of normal or cancer-specific cassette exons. [file 1476-4598-9-230-S10.JPEG]
