# Supplementary material for: Identification of tumor-associated cassette exons in human cancer through EST-based computational prediction and experimental validation
Source: Mol Cancer. 2010 Sep 2;9:230. doi: 10.1186/1476-4598-9-230 (PMC2941758; doi:10.1186/1476-4598-9-230)
Supplement: Additional file 6 — Functional analysis of 46 genes characterized by a normal- or tumoral- specific cassette exon. The file contains enriched biological terms identified with DAVID (Database for Annotation, Visualization and Integrated Discovery) v6.7 (page 1). Most significant terms include "acetylation", "phosphoprotein" and "alternative splicing". An Ingenuity Pathway Analysis, which delivers an assessment of the signalling and metabolic pathways, molecular networks, and biological processes that are most significantly perturbed in a dataset of interest, is also included (pages 2-8). The "Top Networks" section contains the four networks, with a statistically significant score, that could be detected from the input dataset. The network with the highest score (score = 42) is significantly enriched in the following functions: Cell-mediated Immune Response, Cellular Function and Maintenance, Hematological System Development and Function. The report also includes (pages 7 and 8) a graphical representation of two top networks which comprise genes belonging to the network and the relationships among them. Other key components of the IPA Core Analysis, shown in the report, are: "Diseases and Disorders", "Molecular and Cellular Functions" and "Physiological System Development and Function". The number of molecules (i.e. genes) belonging to the different classes and associated p-values are reported. [file 1476-4598-9-230-S6.PDF]

DAVID: Database for Annotation, Vis...

79 chart records

Download File

Sublist	Category	Term	RT	Genes	Count	%	P-Value	Benjamini
	SP_PIR_KEYWORDS	acetylation	RT		22	48,9	5,7E-8	9,1E-6
	SP_PIR_KEYWORDS	phosphoprotein	RT		33	73,3	2,6E-6	2,0E-4
	SP_PIR_KEYWORDS	alternative splicing	RT		31	68,9	8,0E-5	4,2E-3
	UP_SEQ_FEATURE	splice variant	RT		31	68,9	8,4E-5	2,3E-2
	GOTERM_CC_FAT	membrane-bounded vesicle	RT		9	20,0	1,5E-4	2,2E-2
	GOTERM_BP_FAT	generation of precursor metabolites and energy	RT		7	15,6	1,6E-4	6,9E-2
	GOTERM_CC_FAT	vesicle	RT		9	20,0	4,5E-4	3,3E-2
	SP_PIR_KEYWORDS	muscle	RT		3	6,7	4,6E-4	1,8E-2
	GOTERM_CC_FAT	cytoplasmic membrane-bounded vesicle	RT		8	17,8	7,4E-4	3,6E-2
	GOTERM_CC_FAT	actin cytoskeleton	RT		6	13,3	8,8E-4	3,2E-2
	GOTERM_CC_FAT	cytoplasmic vesicle	RT		8	17,8	1,8E-3	5,3E-2
	SP_PIR_KEYWORDS	cytoplasm	RT		17	37,8	2,0E-3	6,0E-2
	GOTERM_CC_FAT	melanosome	RT		4	8,9	2,0E-3	4,8E-2
	GOTERM_CC_FAT	pigment granule	RT		4	8,9	2,0E-3	4,8E-2
	GOTERM_CC_FAT	cytosol	RT		11	24,4	2,9E-3	6,0E-2
	GOTERM_BP_FAT	actin filament-based process	RT		5	11,1	3,7E-3	5,7E-1
	SP_PIR_KEYWORDS	actin binding	RT		3	6,7	3,8E-3	9,5E-2
	SP_PIR_KEYWORDS	glycolysis	RT		3	6,7	4,6E-3	9,8E-2
	GOTERM_BP_FAT	glycolysis	RT		3	6,7	6,9E-3	6,5E-1
	SP_PIR_KEYWORDS	protein biosynthesis	RT		4	8,9	9,1E-3	1,6E-1
	GOTERM_BP_FAT	glucose catabolic process	RT		3	6,7	1,0E-2	6,9E-1
	GOTERM_BP_FAT	hexose catabolic process	RT		3	6,7	1,4E-2	7,3E-1

Completato

IPA® Summary of Analysis

Analysis Name: 46genes - 2010-07-21 03:53 PM
Analysis Creation Date: 2010-07-21
IPA version: 8.6 (Release Date: 2010-05-28)
Content version: 3002 (Release Date: 2010-04-22)

Analysis settings

View

Reference set: Ingenuity Knowledge Base (Genes Only)

Relationship to include: Direct and Indirect

Includes Endogenous Chemicals

Optional Analyses: My Pathways My List

Filter Summary:

Consider only relationships where

data sources = Additional interactions OR Ingenuity Expert Findings OR MicroRNA-mRNA interactions OR Protein-protein interactions

Top Networks

ID	Associated Network Functions	Score
1	Cell-mediated Immune Response, Cellular Function and Maintenance, Hematological System Development and Function	42
2	Cell Death, Cell Cycle, Free Radical Scavenging	31
3	Gene Expression, Cell-To-Cell Signaling and Interaction, Cellular Assembly and Organization	25
4	Genetic Disorder, Neurological Disease, Embryonic Development	2

(c) 2000-2010 Ingenuity Systems, Inc. All rights reserved.

Top Bio Functions

Diseases and Disorders

Name	p-value	# Molecules
Skeletal and Muscular Disorders	1,54E-04 - 4,49E-02	22
Cancer	3,77E-04 - 4,03E-02	22
Genetic Disorder	3,98E-04 - 4,49E-02	32
Metabolic Disease	2,83E-03 - 2,12E-02	4
Cardiovascular Disease	3,06E-03 - 2,42E-02	4

Molecular and Cellular Functions

Name	p-value	# Molecules
Cellular Assembly and Organization	5,46E-05 - 4,78E-02	11
DNA Replication, Recombination, and Repair	5,46E-05 - 4,78E-02	6
Energy Production	1,90E-04 - 4,20E-02	2
Nucleic Acid Metabolism	1,90E-04 - 4,20E-02	3
Small Molecule Biochemistry	1,90E-04 - 4,20E-02	10

Physiological System Development and Function

Name	p-value	# Molecules
Connective Tissue Development and Function	3,06E-03 - 3,31E-02	5
Embryonic Development	3,06E-03 - 1,22E-02	4
Hematological System Development and Function	3,06E-03 - 4,20E-02	4
Lymphoid Tissue Structure and Development	3,06E-03 - 3,02E-02	1
Nervous System Development and Function	3,06E-03 - 1,22E-02	2

(c) 2000-2010 Ingenuity Systems, Inc. All rights reserved.

Top Canonical Pathways

Name	p-value	Ratio
Antigen Presentation Pathway	1,78E-04	3/39 (0,077)
Allograft Rejection Signaling	3,46E-04	3/59 (0,051)
Graft-versus-Host Disease Signaling	3,46E-04	3/49 (0,061)
Autoimmune Thyroid Disease Signaling	3,94E-04	3/61 (0,049)
Virus Entry via Endocytic Pathways	2,29E-03	3/96 (0,031)

Top Molecules

This analysis has no expression values.

(c) 2000-2010 Ingenuity Systems, Inc. All rights reserved.

INGENUITY® SYSTEMS

Top My Lists

Name	p-value	Ratio

Top My Pathways

Name	p-value	Ratio

Top Tox Lists

Name	p-value	Ratio
Cholesterol Biosynthesis	4,78E-02	1/16 (0,062)
Hepatic Stellate Cell Activation	1,02E-01	1/35 (0,029)
Hypoxia-Inducible Factor Signaling	1,93E-01	1/70 (0,014)
VDR/RXR Activation	2,11E-01	1/77 (0,013)
NFκB Signaling Pathway	2,91E-01	1/112 (0,009)

(c) 2000-2010 Ingenuity Systems, Inc. All rights reserved.

INGENUITY® SYSTEMS

Top Tox Functions

Cardiotoxicity

Name	p-value	# Molecules
Cardiac Damage	3,06E-03 - 3,06E-03	1
Cardiac Fibrosis	5,65E-02 - 5,65E-02	1
Cardiac Dilation	9,90E-02 - 9,90E-02	1
Cardiac Necrosis/Cell Death	2,78E-01 - 2,78E-01	1
Cardiac Hypertrophy	3,11E-01 - 3,11E-01	1

Hepatotoxicity

Name	p-value	# Molecules
Liver Hepatomegaly	4,20E-02 - 4,20E-02	1
Liver Steatosis	1,55E-01 - 1,55E-01	1
Liver Cholestasis	1,68E-01 - 1,68E-01	1
Liver Damage	2,37E-01 - 2,37E-01	1

Nephrotoxicity

Name	p-value	# Molecules
Renal Tubule Injury	2,48E-01 - 2,48E-01	1

(c) 2000-2010 Ingenuity Systems, Inc. All rights reserved.

INGENUITY® SYSTEMS

Path Designer Network 1

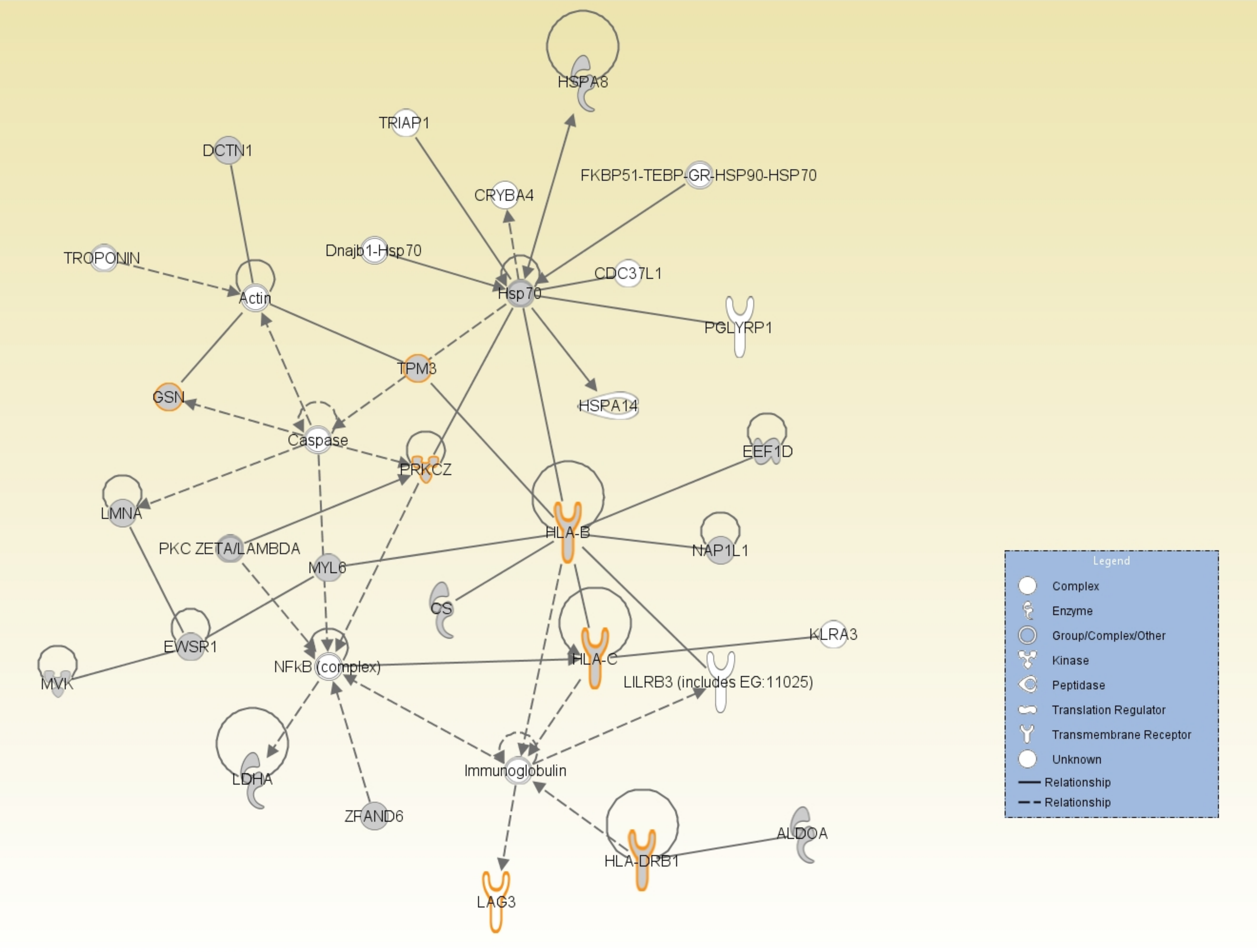

© 2000-2010 Ingenuity Systems, Inc. All rights reserved.

Path Designer Network 2

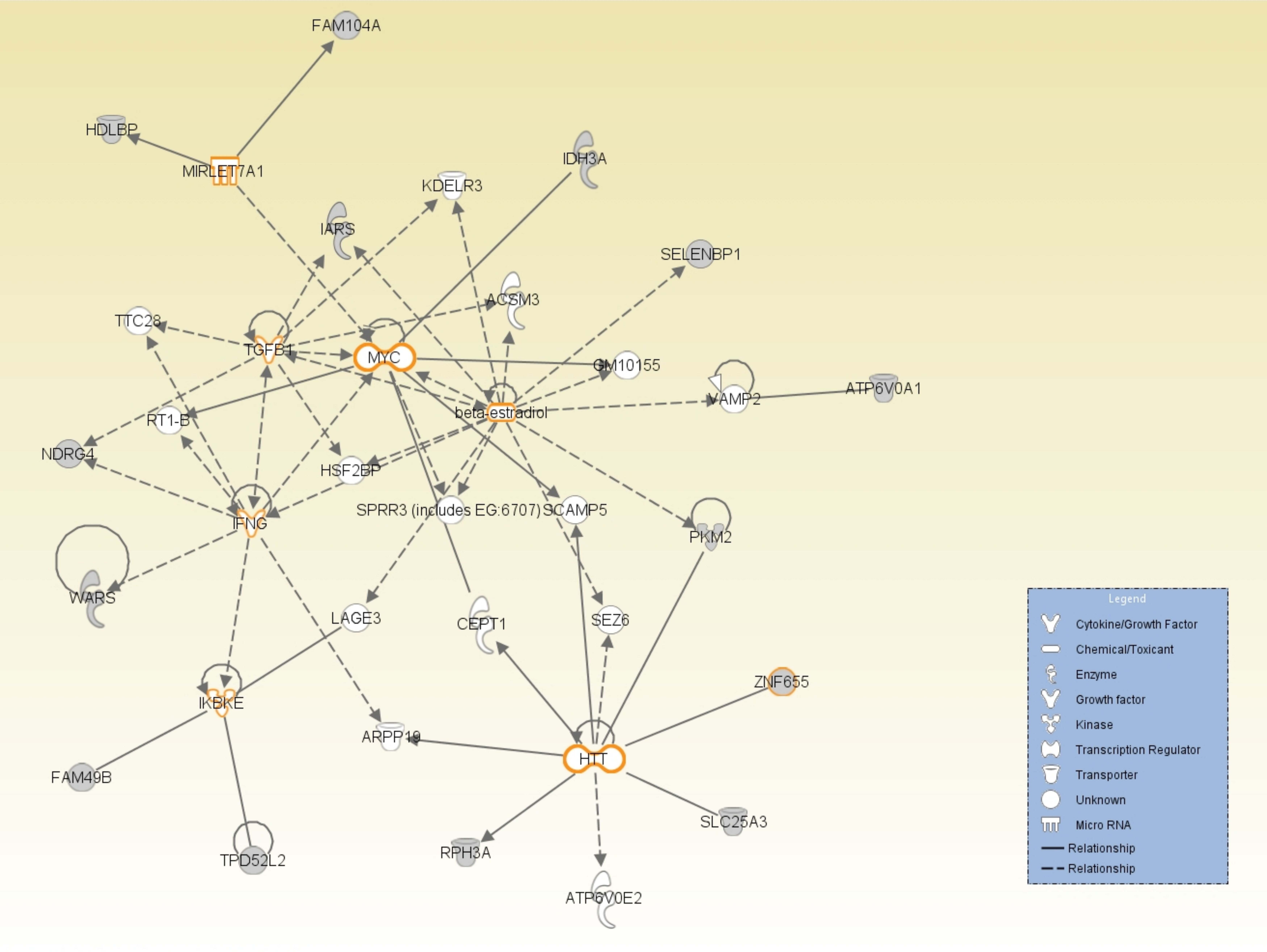

© 2000-2010 Ingenuity Systems, Inc. All rights reserved.
