# Supplementary material for: Identification of tumor-associated cassette exons in human cancer through EST-based computational prediction and experimental validation
Source: Mol Cancer. 2010 Sep 2;9:230. doi: 10.1186/1476-4598-9-230 (PMC2941758; doi:10.1186/1476-4598-9-230)
Supplement: Additionale file 8 — List of predicted normal (A) and tumor (B) -specific cassette exons. The anatomical group, the gene name, the genomic coordinates (NCBI36/hg18), and the unique transcript ID following Riva and Pesole (2009) are reported. [file 1476-4598-9-230-S8.DOC]

**(A) Normal-specific cassette exons**

| **Gene** | **Tissue** | **Cassette Exon** | **Normal TS** | **Cancer TS** |
| --- | --- | --- | --- | --- |
| ATP6V0A1 | CNS | Chr17:37919833-37920004 (172 nt) | 0794e95327:22 | 79f8c2fed1:21 |
| PCNP | CNS | Chr3:102786971-102787045 (75 nt) | 5be4f52a00:5 | 73b7a9c7c1|3 |
| PCNP | CNS | Chr3:102791749-102791804 (56 nt) | 5be4f52a00:5 | 73b7a9c7c1|3 |
| STRADA | CNS | Chr17:59157730-59157787 (58 nt) | 07c9d899fe:13 | 1bd31a27a1:11 |
| STRADA | CNS | Chr17:59154390-59154418 (29 nt) | 07c9d899fe:13 | 1bd31a27a1:11 |
| SELENBP1 | CNS | Chr1:149608104-149608289 (186 nt) | 923bfc79a1:12 | 0bfccde65d:10 |
| SELENBP1 | CNS | Chr1:149608542-149608654 (113 nt) | 923bfc79a1:12 | 0bfccde65d:10 |
| SELENBP1 | CNS | Chr1:149608104-149608654 (551 nt) | eaa3d602e9:5 | 0bfccde65d:10 |
| EWSR1 | CNS | Chr22:28000254-28000271 (18 nt) | 4359e4932b:18 | f6e1400b00:17 |
| UQCC | CNS | Chr20:33435255-33425350 (96 nt) | a34864bc42:10 | cc0bcfd9ec|7 |
| UQCC | CNS | Chr20:33425401-33425473 (73 nt) | a34864bc42:10 | cc0bcfd9ec|7 |
| UQCC | CNS | Chr20:33433135-33433242 (108 nt) | a34864bc42:10 | cc0bcfd9ec|7 |
| UQCC | CNS | Chr20:33425401-33425473 (73 nt) | a34864bc42:10 | 812013da05:7 |
| UQCC | CNS | Chr20:33417774-33417831 (58 nt) | a34864bc42:10 | 812013da05:7 |
| DCTN1 | CNS | Chr2:74454958-74454975 (18 nt) | f14720fd48:33 | 003ad1c263:23 |
| DCTN1 | CNS | Chr2:74452172-74452363 (192 nt) | f14720fd48:33 | 003ad1c263:23 |
| DCTN1 | CNS | Chr2:74451614-74451811 (198 nt) | 962856b814:28 | 003ad1c263:23 |
| DCTN1 | CNS | Chr2:74457377-74457397 (21 nt) | f14720fd48:33 | 003ad1c263:23 |
| DCTN1 | CNS | Chr2:74458635-74458880 (246 nt) | 962856b814:28 | 003ad1c263:23 |
| DCTN1 | CNS | Chr2:74458283-74458361 (79 nt) | 962856b814:28 | 003ad1c263:23 |
| DCTN1 | CNS | Chr2:74458067-74458101 (35 nt) | 962856b814:28 | 003ad1c263:23 |
| DCTN1 | CNS | Chr2:74452172-74452363 (192 nt) | 962856b814:28 | 003ad1c263:23 |
| DCTN1 | CNS | Chr2:74458067-74458101 (35 nt) | f14720fd48:33 | 003ad1c263:23 |
| DCTN1 | CNS | Chr2:74458635-74458880 (246 nt) | 5d67ad0426:32 | 003ad1c263:23 |
| DCTN1 | CNS | Chr2:74453563-74453583 (21 nt) | 63df2edf27:28 | 003ad1c263:23 |
| NDRG4 | CNS | Chr16:57092393-57092482 (90 nt) | 0845809853:15 | cf47127a9b:15 |
| NRSN2 | CNS | Chr20:278282-278476 (195 nt) | 8b962050a5:4 | cbd5244e91|3 |
| IARS | CNS | Chr9:94088639-94088721 (83 nt) | 45a24b7d92:34 | 83d3103025|15 |
| IARS | CNS | Chr9:94087825-94087942 (118 nt) | 45a24b7d92:34 | 83d3103025|15 |
| IARS | CNS | Chr9:94089894-94090013 (120 nt) | 45a24b7d92:34 | 83d3103025|15 |
| ACTN1 | CNS | Chr14:68462028-68462142 (115 nt) | 084e48f4f8:21 | fa5c03a672|16 |
| FAM49B | CNS | Chr8:130985927-130986013 (87 nt) | 8c48e78fac:15 | 6db04283f1:13 |
| CTNNA1 | CNS | Chr5:138244269-138244321 (53 nt) | fb79cba600:16 | a6037d518d:18 |
| CTNNA1 | CNS | Chr5:138244630-138244725 (96 nt) | fb79cba600:16 | a6037d518d:18 |
| TMEM87A | CNS | Chr15:40351553-40351613 (61 nt) | 36f9ebbd7f:20 | f631cfdf3a|19 |
| MVK | CNS | Chr12:108501990-108502134 (145 nt) | da6a51d3cb:11 | 6d352835be|9 |
| RPH3A | CNS | Chr12:111758679-111758690 (12 nt) | ef38b7cfb4:24 | d8bdce07bc:21 |
| ITIH4 | LIV | Chr3:52827313-52827339 (27 nt) | 0975a64cb5:24 | 86d2f7a8fa:21 |
| LDHA | DER | Chr11:18378753-18378868 (116 nt) | dc109ddd7d:7 | 2aa7513c54:8 |
| PLEKHB1 | EYE | Chr11:73044496-73044600 (105 nt) | 820a4dd43d:9 | 7fbe72beb1:7 |
| HLA-C | LYM | Chr6:31347355-31347624 (270 nt) | 8a494cf481:8 | f62556ccd3:8 |
| IDH3A | LYM | Chr15:76236305-76236559 (255 nt) | 72bc9614a1|12 | cf4fe9bd0b:11 |
| HLA-C | MSK | Chr6:31347355-31347624 (270 nt) | 8a494cf481:8 | 97bf7ff0de:5 |
| SLC25A3 | MSK | Chr12:97513342-97513466 (125 nt) | 20d095a928:8 | 74428a1b1c:8 |
| TPD52L2 | MSK | Chr20:61977613-61977672 (60 nt) | 43e3a89b4d:7 | 36120a8743:6 |
| TPM3 | MSK | Chr1:152408405-152408483 (79 nt) | 3c6f350bf4:11 | 604ca5fb15:8 |
| RPS24 | MSK | Chr10:79467729-79467746 (18 nt) | 5ca871b326:7 | 5073f86fae:6 |
| RPS24 | MSK | Chr10:79467729-79467746 (18 nt) | 5ca871b326:7 | bc870530d8:6 |
| MYL6 | PLA | Chr12:54840677-54840721 (45 nt) | 726470fdc3:7 | 12564efe8a|8 |
| HLA-B | RES | Chr6:31346829-31347104 (276 nt) | 5907c2f650:9 | 8aff531913:10 |
| HLA-B | RES | Chr6:31345966-31346241 (276 nt) | 5907c2f650:9 | 8aff531913:10 |
| PKM2 | TES | Chr15:70300563-70300679 (117 nt) | c85662e674:12 | 3bd3164e66:11 |
| FAM104A | TES | Chr17:68720412-68720474 (63 nt) | 1a5980d1d6:5 | c8e1096b98:3 |
| ALDOA | TES | Chr16:29985707-29985860 (154 nt) | 43fdf89d24:10 | 719b1c0910:9 |
| ALDOA | TES | Chr16:29985707-29985860 (154 nt) | 43fdf89d24:10 | c01b216256:14 |
| ZFAND6 | TES | Chr15:78177813-78177975 (163 nt) | 7c71e3e7fc:7 | 595c8358f9:6 |

**(B) Tumoral-specific cassette exons**

| **Gene** | **Tissue** | **Cassette Exon** | **Normal TS** | **Cancer TS** |
| --- | --- | --- | --- | --- |
| CS | CNS | Chr12:54971682-54971844 (163 nt) | b287d00abc:11 | 4139aec2b8|12 |
| METT10D | CNS | Chr17:2325285-2325343 (59 nt) | 18dce49ff6|5 | 60c837e71c:10 |
| DCTN1 | CNS | Chr2:74464388-74464441 (54 nt) | 5d67ad0426:32 | 003ad1c263:23 |
| ZNF655 | CNS | Chr7:98997927-98998053 (127 nt) | 0d603b7120:3 | a4b15a0c82:5 |
| ZNF655 | CNS | Chr7:98997927-98998053 (127 nt) | 0877d416af:4 | a4b15a0c82:5 |
| HLA-DRB1 | CNS | Chr6:32656501-32656611 (111 nt) | 35ba5672cd:6 | 40ce118f49:6 |
| HLA-DRB1 | CNS | Chr6:32656002-32656025 (24 nt) | 35ba5672cd:6 | 40ce118f49:6 |
| YPEL5 | CNS | Chr2:30224615-30224911 (297 nt) | cd85ca1097:3 | 2832aba0dc:4 |
| YPEL5 | CNS | Chr2:30224615-30224833 (219 nt) | cd85ca1097:3 | 69beb7ad30:4 |
| HDLBP | CNS | Chr2: 241857042-241857383 (342 nt) | 43779ed314:29 | 61e497448a:29 |
| HDLBP | CNS | Chr2: 241857042-241857383 (342 nt) | 90db9945ad:28 | 61e497448a:29 |
| GSN | KID | Chr9:123102155-123102225 (71 nt) | 23f79a833f:18 | 053bd62bec:20 |
| LMNA | DER | Chr1:154371602-154371727 (126 nt) | b44eb3f122:6 | 9afaaf1b84:10 |
| LMNA | DER | Chr1:154372316-154372536 (221 nt) | b44eb3f122:6 | 9afaaf1b84:10 |
| LMNA | DER | Chr1:154372629-154372851 (223 nt) | b44eb3f122:6 | 9afaaf1b84:10 |
| LMNA | DER | Chr1:154373336-154373443 (108 nt) | b44eb3f122:6 | 9afaaf1b84:10 |
| LMNA | DER | Chr1:154373528-154373647 (120 nt) | b44eb3f122:6 | 9afaaf1b84:10 |
| LMNA | DER | Chr1:154373336-154373647 (312 nt) | b44eb3f122:6 | 3e79ca7b23:11 |
| HLA-C | DER | Chr6:31346829-31347104 (276 nt) | 97bf7ff0de:5 | 8a494cf481:8 |
| HSPA8 | DER | Chr11:122434170-122434402 (233 nt) | 59326c4c18:8 | d10c1db847:9 |
| EEF1D | EYE | Chr8:144745960-144745973 (14 nt) | 53cdcbab38:8 | 687088b20e:10 |
| EEF1D | EYE | Chr8:144742304-144743394 (1091 nt) | 53cdcbab38:8 | 687088b20e:10 |
| HLA-B | LYM | Chr6:31430389-31430421 (33 nt) | 856a65a1cb:8 | a13be7544a:8 |
| HLA-B | LYM | Chr6:31430389-31430421 (33 nt) | 5907c2f650:9 | a13be7544a:8 |
| HLA-B | LYM | Chr6:31430235-31430282 (48 nt) | 856a65a1cb:8 | a13be7544a:8 |
| HLA-B | LYM | Chr6:31430235-31430282 (48 nt) | 5907c2f650:9 | a13be7544a:8 |
| HLA-B | LYM | Chr6:31429915-31430052 (138 nt) | 856a65a1cb:8 | 8aff531913:10 |
| HLA-B | LYM | Chr6:31429915-31430052 (138 nt) | 5907c2f650:9 | 8aff531913:10 |
| WARS | LYM | Chr14:99910226-99910355 (130 nt) | 06e5592e3c:11 | 78222216c3:13 |
| NAP1L1 | MSK | Chr12:74748956-74749041 (86 nt) | b961691640:15 | 912e61d745:15 |
| NAP1L1 | MSK | Chr12:74747418-74747520 (103 nt) | b961691640:15 | 912e61d745:15 |
| SLC25A3 | MSK | Chr12:97513636-97513757 (122 nt) | 20d095a928:8 | 74428a1b1c:8 |
| PRKCZ | PLA | Chr1:2056333-2056385 (53 nt) | 4348c85d6f|17 | f76ced1f5c|17 |
| HLA-B | RES | Chr6:31430389-31430421 (33 nt) | 5907c2f650:9 | a13be7544a:8 |
| HLA-B | RES | Chr6:31430235-31430282 (48 nt) | 5907c2f650:9 | a13be7544a:8 |
| HLA-B | RES | Chr6:31429915-31430052 (138 nt) | 5907c2f650:9 | 8aff531913:10 |
| RAN | RES | Chr12:129923082-129923127 (46 nt) | 1d2a09d586:6 | 3c125d596c:7 |
| POMT1 | TES | Chr9:133369397-133369648 (152 nt) | fd01629184:18 | 7df96a1ad3:20 |
| POMT1 | TES | Chr9:133371322-133371428 (107 nt) | fd01629184:18 | 7df96a1ad3:20 |
